# A general soft-enveloping strategy in the templating synthesis of mesoporous metal nanostructures

**DOI:** 10.1038/s41467-018-02930-9

**Published:** 2018-02-06

**Authors:** Jixiang Fang, Lingling Zhang, Jiang Li, Lu Lu, Chuansheng Ma, Shaodong Cheng, Zhiyuan Li, Qihua Xiong, Hongjun You

**Affiliations:** 1 Key Laboratory of Physical Electronics and Devices of the Ministry of Education, School of Electronic and Information Engineering, Xi’an Jiaotong University, Xi’an, Shannxi 710049 China; 2 School of Microelectronics, Xi’an Jiaotong University, Xi’an, Shannxi 710049 China; 3 College of Physics and Optoelectronic Engineering, South China University of Technology, Guangzhou, 510640 China; 4 Division of Physics and Applied Physics, School of Physical and Mathematical Sciences, Nanyang Technological University, Singapore, 637371 Singapore

## Abstract

Metal species have a relatively high mobility inside mesoporous silica; thus, it is difficult to introduce the metal precursors into silica mesopores and suppress the migration of metal species during a reduction process. Therefore, until now, the controlled growth of metal nanocrystals in a confined space, i.e., mesoporous channels, has been very challenging. Here, by using a soft-enveloping reaction at the interfaces of the solid, liquid, and solution phases, we successfully control the growth of metallic nanocrystals inside a mesoporous silica template. Diverse monodispersed nanostructures with well-defined sizes and shapes, including Ag nanowires, 3D mesoporous Au, AuAg alloys, Pt networks, and Au nanoparticle superlattices are successfully obtained. The 3D mesoporous AuAg networks exhibit enhanced catalytic activities in an electrochemical methanol oxidation reaction. The current soft-enveloping synthetic strategy offers a robust approach to synthesize diverse mesoporous metal nanostructures that can be utilized in catalysis, optics, and biomedicine applications.

## Abstract (web summary)

Metal species are highly mobile within mesoporous silica, making it difficult to template growth of metallic nanocrystals inside the channels. Here, the authors introduce a solid-liquid-solution interfacial strategy to suppress migration of the metal species, achieving control over a variety of mesostructured nanomaterials.

## Introduction

Since the discovery of ordered mesoporous silicas in the 1990s^1–3^, ordered mesoporous materials have attracted much interest owing to their wide range of applications in biosensors, surface-enhanced spectroscopy, separation, drug delivery, catalysis, and fuel cells, which cannot be achieved by other compositions or structures^4–8^. These fascinating applications are inherent to metal frameworks with high porosities, large areas per unit volume, tunable pore sizes, narrow pore size distributions, high electroconductivities, and excellent activity–structure relationships. To date, mesoporous metals in bulk, thin film and powder forms have been exploited by means of electrochemical dealloying, soft-templating or hard-templating, and solution phase approaches^9–13^. Generally, soft-templating and hard-templating methods have achieved good pore size and structural control. However, the obtained morphologies of mesoporous metals have been limited to mainly powders with irregular morphology or films on conductive substrates^14–16^. These limitations are very serious for the further development of these metals since in some areas such as optical spectroscopy, catalysis, and biomedicine, the unambiguous identification of nanoparticle (NP) properties requires monodisperse NPs with a well-defined particle size, shape, composition, and crystal structure to avoid averaging effects^17,18^. In addition, the reliable performance in some applications requires consistent properties that are only possible with monodisperse NPs^19,20^.

**Fig. 1:**
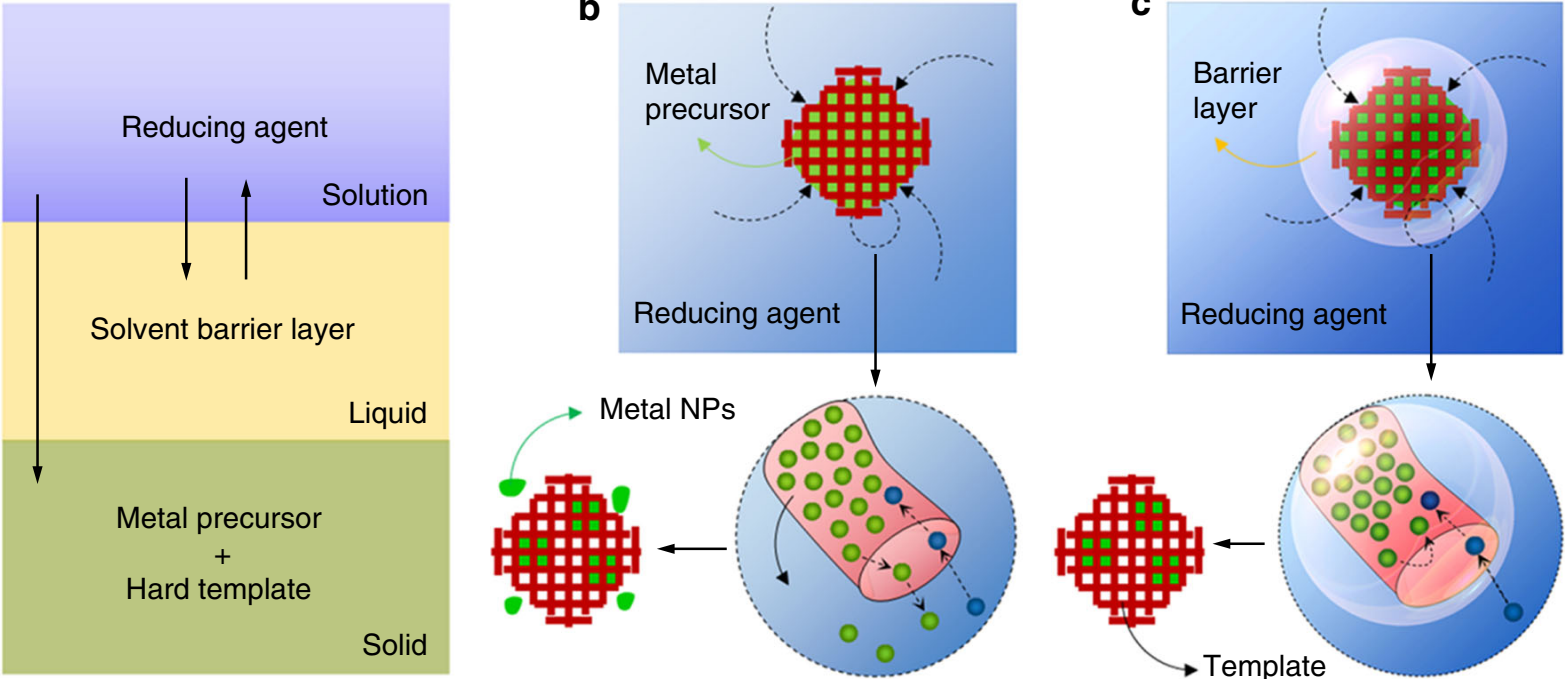
The schematic illustrations of soft-enveloping strategy. **a** The proposed SLS (solid–liquid–solution) three phase interface, in which a solvent (“liquid”) that cannot dissolve metal precursors was used as a surface coating and a barrier layer to prevent metal species from migrating to the outside of the mesoporous channels (“solid”). Next, the transfer of the reducing agent ions (“solution”) across the solvent and then the reduction of the metal precursor at the interface of the “solid” and “solution”. **b** The present challenge is that the final product mixes with the morphologies containing the bulk particles. **c** The current soft-enveloped SLS interface reaction protocol

To date, although a variety of routes have been developed to synthesize ordered mesoporous metals and metal oxides using mesoporous silica or carbon as a hard template^21^, in comparison with metal oxides, the synthesis of monodispersed ordered mesoporous metals (particularly in gold and silver) with well-controlled sizes and shapes has not yet been conducted by such approaches^22,23^. Only the preparation of mesoporous metals such as platinum, osmium and palladium has been reported^15,24,25^. In almost all the cases that utilize hard-templates, nanostructured Au materials have only been used as spherical NPs, nanosheets and nanowires, without 3D mesoporous structures^26–28^. This is partially because Au and Ag have relatively faster deposition rates than does Pt within the limited space of the mesoporous templates; these elements tend to grow rapidly along the dominated channels, and their growth in the micropores is insufficient^29^. In addition, negatively charged metallic species (ions, atoms, and cluster) have high mobilities inside mesoporous silica and thus prefer to migrate to the outside of the mesoporous silica during a reduction process, resulting in the formation of bulk particles and mixed morphologies (Fig. 1b)^22^. Therefore, in recent years, various protocols such as the surface functionalization of host silica^30^, ion-exchange^31^, CTA-AuBr_4_ complex formationss^27^, glow discharge plasma reduction^32^, and metal-block copolymer procedure^33^, have been attempted to suppress the migration of metallic species. Understanding the filling, diffusion, and growth processes inside mesoporous silica during nanocasting and reduction is critical to controlled synthesis and further applications^34–36^.

Here, we exploit an effective strategy to prevent the migration of metal species to the outside of mesoporous silica, and successfully synthesize monodispersed nanostructures with well-defined sizes and shapes, including Ag nanowires, 3D mesoporous Au, AuAg alloys, and Pt networks, using a chemical reduction process (Fig. 1c). This strategy is based on a soft-enveloping function on the surface of the metal precursor immersed in silica and a phase transfer mechanism that occurs at the interface of the solid–liquid solution (SLS) phase (Fig. 1a). Specifically, a solvent (“liquid”) that cannot dissolve metal precursors is used as a barrier layer and is designed to prevent metal species from migrating to the outside of mesoporous channels (“solid”). During the following reduction stage, a phase transfer process occurrs spontaneously across the interface of the “solution” (containing the reducing agent ions) and the “liquid” and leads to the reduction at the interface between the “solid” and “solution” phases. We believe the current methodology provides a simple and robust way to synthesize a variety of mesoporous NPs in metals.

## Results

### Structural characterization and controls

**Fig. 2:**
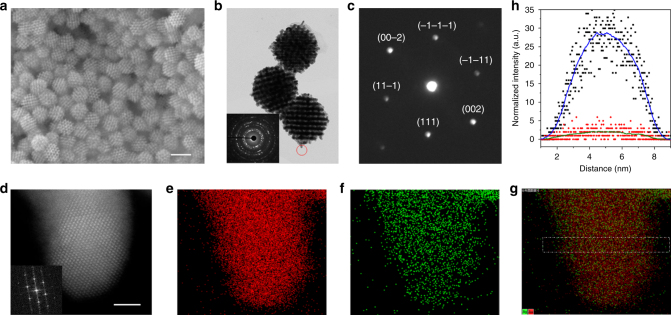
The characterization analysis of the 3D ordered mesoporous AuAg networks. **a**, **b** The typical SEM and TEM images of the 3D ordered mesoporous AuAg networked structures synthesized via the proposed soft-enveloped SLS interface reaction. **c** The [1–10] zone axis nanobeam electron diffraction pattern, displaying typical {200} and {111} Bragg reflections of fcc, Au, or Ag. **d**–**g** Representative HAADF-STEM image in combination with EDX element maps. Inset in **d** is the fast Fourier transformation (FFT) patterns of the STEM images. **h** The composition line profiles obtained by EDX with an electron beam (~1.5 Å in spot size) scanning across the region marked in **g**. The scale bars in **a** and **d** are 100 and 2 nm, respectively

We chose a 3D mesoporous Au system as an example to demonstrate the validity and advantages of our method in yielding high-quality mesostructures in metals. KIT-6 (orderly mesoporous silica) was selected as a hard template to synthesize the 3D mesoporous Au networks. Hexane (or dichloromethane) and 1,1,3,3-tetramethyldisiloxane (TMDS) (or dimethylaminoborane (DMAB) or butylamine) were used as the barrier layer and reducing agent, respectively. Since both hexane and dichloromethane cannot dissolve HAuCl_4_ and AgNO_3_, the reduction agent (e.g., DMAB, butylamine, or TMDS) may diffuse into the KIT-6 matrix through the barrier layer to react with HAuCl_4_ and AgNO_3_ (Fig. 1a). Thus, by means of the co-reduction of HAuCl_4_ and AgNO_3_, single component Au and a binary AuAg alloy can be synthesized with a Ag content from 0 to 12 wt% (determined using inductively coupled plasma atomic emission spectroscopy (ICP-AES)). Figure 2a shows the scanning electron microscopy (SEM) image of typical 3D mesoporous AuAg nanostructures after the removal of the silica template. The mesoporous AuAg NPs display a monodispersed ordered porous structure with good uniformity and a narrow particle size distribution with an average diameter of ~100 nm and a standard deviation of ~4.66 (Supplementary Fig. 1). A high-magnification SEM image demonstrates that the mesoporous AuAg NPs have a polyhedral morphology, probably a cubic shape (Supplementary Fig. 2). The HRTEM images in Supplementary Fig. 3 show that large-sized mesopores approximately 14–15 nm are created and correspond to the total volume of the wall thicknesses plus the pore size of KIT-6. This square-shaped network structure indicates that the mesoporous AuAg NPs replicate a one-sided pore system in a bicontinuous structure^25,37^. Figure 2b shows a transmission electron microscopy (TEM) image of the AuAg networked nanostructure. The selected-area electron diffraction (SAED) pattern inserted in Fig. 2b confirms that the individual AuAg NPs are polycrystalline. While the diffraction pattern (Fig. 2c) obtained within a small region (e.g., the red circle in Fig. 2b) using a nanobeam (~1.5 nm in spot size) displays the typical features of a single crystal, and the reflection spots can be indexed as the Bragg reflections of a Au or a Ag face centered cubic (fcc) structure.

The phase structure and composition of the mesoporous AuAg NPs have been further examined at atomic level by means of aberration-corrected, high-angle annular dark-field scanning transmission electron microscopy (HAADF- STEM) in combination with energy dispersive X-ray spectroscopy (EDX), as shown in Fig. 2d–g. Figure 2d shows representative HAADF-STEM image of the mesoporous AuAg NPs. Figure 2e and f show the EDX composition maps of Au and Ag, respectively, within the region of Fig. 2d. The Au (red) vs. Ag (green) composite image (Fig. 2g) displays that the Au and Ag element are highly intermixed. The sketched trend lines (Fig. 2h) obtained by scanning the e-beam across a crystal tip are almost identical across the tip, indicating the homogeneous alloy nature of the mesoporous AuAg NPs^38^. This statement is further demonstrated by a statistical analysis (Supplementary Figs. 4a–e) of the elemental distributions for different regions of the AuAg NPs and the composition line profiles obtained by EDX with an electron beam diameter of ~1.5 Å.

Further evidence comes from X-ray photoelectron spectroscopy (XPS) data, which are shown in Supplementary Figs. 5a–c and Supplementary Figs. 6a–d. From Supplementary Fig. 5a–c, it can be observed that, compared with the standard binding energies of Au and Ag, the peaks of Ag 4d_5/2_ and Ag 4d_3/2_ show an obvious shift. Moreover, with an increase in the Ag ratio from 0 to 12 wt%, the Au 4f_7/2_ and Au 4f_5/2_ peaks shift to higher binding energies gradually (Supplementary Fig. 6a, b). The XPS peak shifting can be related to the perturbation in the electronic interaction between the Au and Ag atomic orbitals and in turn to alloy formation^39^. The X-ray diffraction (XRD) pattern of mesoporous AuAg NPs is shown Supplementary Fig. 7. The four diffraction peaks are consistent with those of Au or Ag metals with a fcc structure that correspond to the (111), (200), (220), and (311) planes. Based on the HAADF-STEM, EDX, XPS, and XRD data, the controlled phase structure of bimetallic AuAg alloy NPs with a composition of ~12 wt% Ag can be obtained by adjusting the feeding ratio of AgNO_3_^40,41^_._

In addition to the phase structure control, the current soft-enveloping strategy also demonstrates also a robust capability in shape or morphology control as shown in Supplementary Figs. 8–10. A variety of Au nanostructures, such as NPs, dog-bone shapes, multiple pods, and mesoporous structures (Supplementary Figs. 8–19) with tunable sizes can be successfully synthesized by controlling the growth time of the formation of the Au structure. Supplementary Figs. 8a–f show the schematic illustration for the synthesis of well-defined nanostructured Au NPs from a KIT-6 silica template. During the early stage of Au growth within 10 min, spherical Au NPs, dog-bone shapes, and multiple pods are obtained (Supplementary Figs. 9a and 8a). With an increase in the reduction time from 30 min to 24 h, the Au NPs display an ordered mesoporous morphology with increasing sizes from ~50 nm (Supplementary Figs. 8c and 9b) to ~100 nm (Supplementary Fig. 9c) and ~150 nm (Supplementary Fig. 9d). To investigate the morphologies of the products under a variety of reaction conditions, we have also studied the influence of reactant concentrations. At relatively low or high HAuCl_4_ concentrations, e.g., 0.5, 10, and 100 mM, ordered mesoporous Au nanostructures with different particle sizes can still be obtained (Supplementary Figs. 10a–c). It is revealed that the current synthetic strategy has a strong capability to tune the morphologies and sizes of the products.

### Formation mechanism

**Fig. 3:**
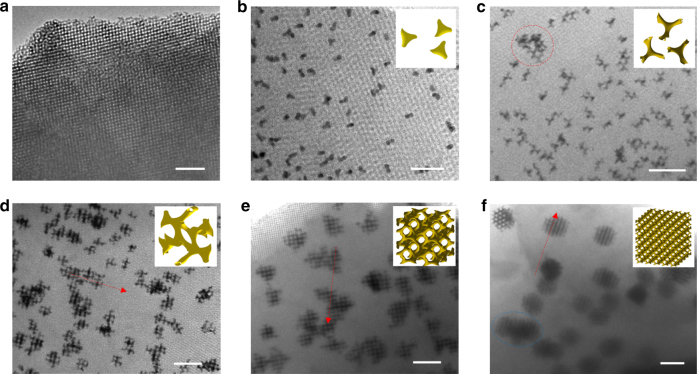
TEM images of a series of Au nanostructures/silica composites. Different morphologies and sizes is obtained by changing the reduction time. **a** 0 min, before the reduction reaction, displaying the Au precursor distributed homogenously in the ordered mesoporous silica matrix. **b** 2 min, showing the Au nanoparticles with a wide size distribution. **c** 8 min, the Au NPs show a structure of three-pods. **d** 15 min, the Au NPs with networked morphology has been formed. **e-f** 2 and 6 h, 3D mesoporous Au networks, the red arrows indicate that the Au NPs deposited within the same single mesostructural Ia3d domain of the original KIT-6. The scale bars in **a**, **b**, and **c**–**f** are 100, 50, and 100 nm, respectively

To understand the growth processes of mesoporous Au NPs within a KIT-6 matrix, the mesoporous Au/silica composites before silica removal were carefully investigated by TEM. Figure 3 demonstrates a series of Au nanostructures/silica composites with different morphologies and sizes prepared by changing the reduction time, i.e., 0, 2, 8, 15 min, 2 and 6 h. Initially, the Au precursor is distributed homogenously in the ordered mesoporous silica matrix (Fig. 3a and Supplementary Fig. 11). During the very early stage of the reduction reaction, several Au NPs with sizes from less than 10 to ~20 nm are observed clearly (Fig. 3b and Supplementary Fig. 12). During the subsequent growth, three pods or multiple pods and even some network clusters (circled in red in Fig. 3c) can be formed. In this situation, the sizes of multiple pods and clusters are still distributed in a wide range of 20–50 nm. As the reaction proceeds, i.e., after 15 min, small-sized mesoporous Au networks are obtained, displaying a size of approximately 50–70 nm (Fig. 3d). When the reduction time approaches to 2 and 6 h (Fig. 3e, f, respectively), the morphologies of the mesoporous Au networks are not changed, but the average particle sizes reach approximately 80 and 100 nm, respectively. Based on the further observations in Fig. 3, the Au NPs during the early stage are distributed over the entire silica matrix and isolated from each other. The initially formed Au networks (Fig. 3d) displayed a ‘**#**’-like structural feature, which contributes to the final formation of polyhedral shapes (Fig. 3e, f). In addition, during the growth of the mesoporous Au networks, the Au NPs are deposited within the same single mesostructural Ia3d domain of the original KIT-6 (red arrows in Fig. 3e, f) materials, revealing that a well-controlled replication process of the mesoporous silica has been successfully realized.

According to the above results, the growth mechanism of the 3D mesoporous Au network is proposed and schematically illustrated in Supplementary Fig. 13. The formation of mesoporous Au networks that originate from homogenously distributed Au precursors within a limited space (mesoporous channels) can be described by the classical LaMer curve^42^. These networks consist of three distinct periods: (i) an induction period, (ii) a nucleation period, and (iii) a growth period of Au multiple pods and the mesoporous network. During the induction period, Au^3+^ ions are reduced to Au^0^ atoms. According to time-dependent growth feature of the Au networked nanostructures as shown in Fig. 3 and Supplementary Figs. 9 and 10, the reduced Au atoms are uniformly distributed over the entire silica matrix^25^. Following the induction period, Au atoms start to nucleate via a self- (or homogeneous) nucleation process once the concentration of Au atoms exceeds the supersaturation point. After the nucleation period, according to the in situ UV spectral measurements (Supplementary Figs. 14a and b) and the uniform particle size features shown in Fig. 3, no additional “secondary nucleation” can occur, and most of the reaction time is spent during the growth process^25^. These statements are nicely supported by the following: (i) The density of the Au NPs, multiple pods and mesoporous networks (i.e., the number of Au particles per unit area) do not increase as the growth time is increased (Fig. 3). The slight discrepancy of density may be caused by a bias in the supply of Au sources among the silica matrix. (ii) No small Au clusters can be observed near the mesoporous Au networked NPs (Fig. 3). (iii) The in situ UV spectroscopy measurements can also reveal that, after a very early stage within 60 s, the Au cluster peaks at approximately 400 nm do not appear again during the following growth period (Supplementary Fig. 14b), indicating that no new clusters or nuclei can be continuously formed^43,44^. During the growth period, the Au ions and atoms diffuse from the associated space to each nucleus (i.e., the Voronoi cell)^25^ by tracing the channels of the KIT-6 template, and finally contribute to the growth of the Au nanostructures, including multiple pods and mesoporous network morphologies. In this case, the formed instable nuclei may dissolve into the solution and support the growth process again; thus resulting in the formation of a mesoporous Au networked structure with uniform sizes. In addition, since we are not equipped with a real-time TEM observation capability, we cannot exclude the importance of the aggregation of small NPs (e.g., less than the diameter of the KIT-6 channels). While we believe in present system, the growth of Au networks is an atom-mediated process.

**Fig. 4:**
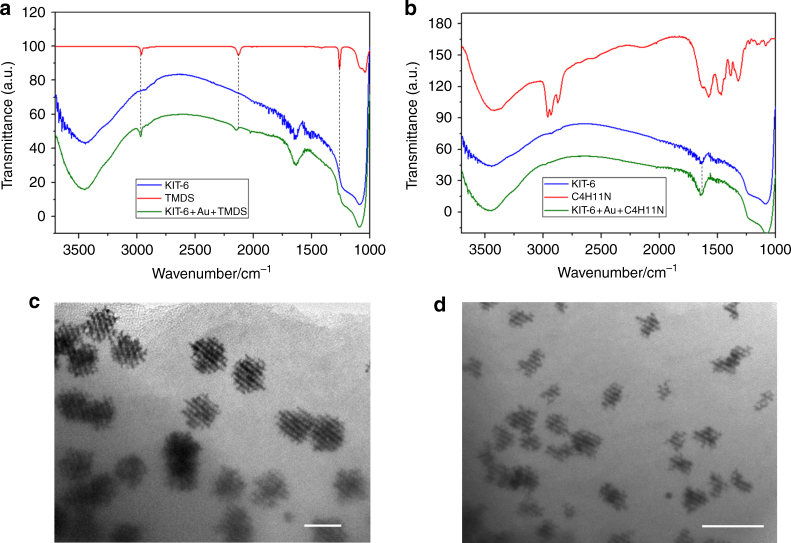
The influences of the reduction agent and solvent barrier layer. **a** FT-IR spectra of the KIT-6 mesoporous template, TMDS reduction agent, and Au compounds. **b** FT-IR spectra of the KIT-6 mesoporous template, butylamine reduction agent, and Au compounds. **c** 3D mesoporous Au networks/silica compounds using butylamine as the reduction agent and hexane as the solvent (scale bar, 100 nm). **d** 3D mesoporous Au networks/silica compounds using TMDS as the reduction agent and dichloromethane as the solvent (scale bar, 200 nm)

In the current soft-enveloping synthetic strategy, hexane and TMDS are mainly selected as the barrier layer solvent and reduction agent, respectively, to avoid the migration of metal species to the outside of the mesoporous silica. In the absence of a barrier layer (“liquid”) in the synthetic sysem (Fig. 1a), some bulk particles without an ordered mesoporous structure can obviously be observed because of Au growth on the outer surface of the mesoporous silica template, as shown in the TEM (Supplementary Fig. 15a) and SEM images (Supplementary Fig. 16a). However, with a barrier layer, the Au species can be effectively controlled to grow inside the mesoporous silica (Supplementary Figs. 15b and 16b). The non-polar TMDS reducing agent may react with the polar silica surfaces and hence suppress the migration of the Au species. This reaction is supported by the FT-IR spectra shown in Fig. 4a, where the peaks located at 2129 cm^−1^ (Si–H stretching) and 2961 cm^−1^ (CH_3_ symstretching) are observed after the reaction. However, the further comparison experiments indicate that the soft-enveloping barrier layer may also play a crucial role to prevent metal species from migrating to the outside of the mesoporous channels. If TMDS is replaced by other reduction agents (e.g., non-polar dimethylaminoborane (DMAB) or polar butylamine), or the soft-enveloping solvent layer was replaced by other solvents (e.g., dichloromethane), then the networked Au nanostructures can be obtained and no obvious bulk particles that have grown outside of silica channels can be observed (Fig. 4c, d and Supplementary Figs. 17–19). Particularly, the FT-IR spectra indicate that the polar butylamine reduction agent does not demonstrate a remarkable reaction between the silica surface and butylamine (Fig. 4b). However, in this system, the well-controlled Au network may still be obtained, further supporting the validity of the current soft-enveloping synthetic strategy.

### Catalytic property

**Fig. 5:**
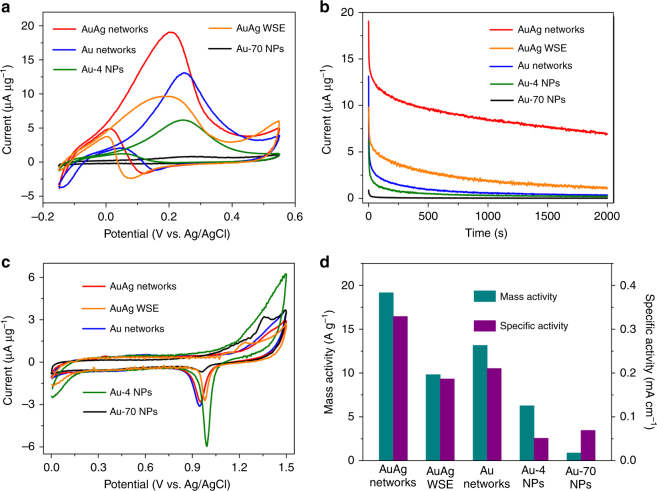
The electrochemical performance. **a** Cyclic voltammograms of the electro-methanol oxidation, **b** chronamperograms, **c** oxide stripping curves, and **d** histograms of the mass activity and specific activity of the AuAg networks, Au networks, Au-4 NPs, and Au-70 NPs. The cyclic voltammograms were performed in 0.5 M KOH and 2 M CH_3_OH at a scan rate of 10 mV s^−1^. The oxide stripping was measured in a 0.5 M H_2_SO_4_ solution at a scan rate of 10 mV s^−1^

Because of the novel 3D framework structure, the high specific surface area, high internal porosity, and rough surface of the mesoporous Au networked nanostructures demonstrate diverse outstanding properties that can be utilized in spectral signal enhancement, controlled drug delivery and cancer combination therapy, and catalysis. To evaluate the catalytic activity of the material, we measured the electrochemical oxidation of methanol as a model reaction for the 3D mesoporous Au and AuAg networks (Fig. 5). For comparison, six kinds of Au NPs with diameters of approximately 3, 4, 5, 6, 20, and 70 nm (as shown in Supplementary Fig. 20a–f and Supplementary Fig. 21), were synthesized and investigated. As shown in Supplementary Fig. 22a–c, among these Au NPs, 4 nm-sized Au NPs show a better mass activity, and the 70 nm-sized Au NPs display a better specific activity. Thus, 4 nm and 70 nm Au nanoparticles (denoted as Au-4 and Au-70 NPs) are selected as references in the study of the Au and AuAg network catalytic property for the methanol oxidation reaction (MOR). In addition, the catalytic property of AuAg products (Supplementary Fig. 15a) synthesized without the soft-enveloping strategy (denoted as AuAg WSE) was also measured as a comparison.

From Fig. 5a, one can see that Au-4 NPs and Au-70 NPs exhibit peak currents for methanol oxidation at 6.2 and 0.9 μA μg^−1^, respectively. While the AuAg networks show much higher peak current with a value up to 19.2 μA μg^−1^, which is 3.1 times that of Au-4 NPs, and 24 times that of Au-70 NPs. Moreover, the peak potentials for methanol oxidation are 0.20, 0.20, 0.25, 0.26, and 0.27 V (Fig. 5a), revealing that methanol oxidation can be easier with the AuAg, and Au networked catalysts and exhibit lower electro-oxidation potentials than those of the Au-4 and Au-70 NPs. The rate of surface poisoning for the five catalysts was evaluated and shown in Fig. 5b. As in previous reports^45,46^, the lagging decreasing rate and the highest current during the entire chronoamperometry process (∼2000 s) are observed for the AuAg networked catalysts, indicating that the electrocatalytic stability of the AuAg networks for the methanol oxidation reaction (MOR) is higher than that of the AuAg WSE, Au networks, Au-4 NPs, and Au-70 NPs. Considering the electrochemically active surface areas (ECSAs) (Fig. 5c) and the mass, both the Au and AuAg networks show greatly improved specific activity and mass activity (Fig. 5d). Specifically, the AuAg networks possess a very high catalytic activity, which is 7 and 5 times higher in specific activity and 3 and 24 times higher in mass activity than that of the Au-4 and Au-70 NPs, respectively (Fig. 5d). Table 1 summarizes the specific activities for several Au and Au alloy catalysts reported in the literature^13,47–53^. Different scanning rates have been used in different studies, and normally a higher current can be obtained with a higher scan rate^54^. Thus, considering the test conditions (i.e., lower scan rate, 10 mV s^−1^, was used in our measurements), our AuAg alloy networks show greatly improved catalytic property compared with that of the Au or AuAg catalysts reported in the literature. In addition to the high catalytic activity and stability, the AuAg networks also show a greatly enhanced durability compared with that of the Au NPs (Supplementary Figs. 23a–c and Supplementary Discussion).

**Table 1:** Specific activity of the Au and AuAg catalysts for the MOR reported in the literature

Types of Au or AuAg catalysts	Measurement conditions^a^	Specific activity^b^	Ref.
AuAg networks	10, 2, 0.5	0.33	This work
AuNPs/LDH/GC electrode	50, 0.5, 0.25	0.11	[48]
Au NPs on vitreous carbon	50, 0.1, 0.1	0.12	[49]
Gold dendrite	10, 1, 0.1	0.2	[50]
Mesoporous Au film	20, 2, 0.1	0.2	[51]
De-alloyed Au NFs	10, 1, 0.1	0.25	[52]
Nanoporous gold	10, 2, 0.5	0.09	[53]
Nanoporous gold bowls	20, 2, 0.5	0.12	[54]
Hollow nanoporous gold	20, 2, 0.5	0.19	[13]

^a^ Scanning speed (mV s^−1^), CH_3_OH and KOH concentrations (M)

^b^ mA cm^−2^

Referring to Fujita’s group and Ling’s group reports^9,13^, we may attribute the superior catalytic performance of the AuAg networks to the high density of the low coordinated atoms, atomic steps and kinks on the curved surface of networked NPs, and the high-index facets (Supplementary Fig. 27), which are formed by the limited growth in the narrow spaces of the mesoporous template. In fact, the obtained atomic structure of the AuAg networks is quite similar to the structure of nanoporous gold prepared via a dealloying process^13^. A more detailed discussion regarding catalytic properties can be seen in the Supplementary Information (Supplementary Figs. 24–26 and Supplementary Discussion).

### Generalization of the current strategy in other systems

The current synthetic strategy can effectively avoid diffusion of metal species to the outside of the mesoporous channels; thus, metallic nanostructures within the mesoporous templates are obtained with controlled shape or morphology. In fact, this protocol may also be easily extended to rather general systems. As mentioned above, we have obtained similar Au mesoporous structures with well-controlled morphology by means of various reduction agents such as TMDS, DMAB and butylamine, and barrier layer solvents, such as hexane and dichloromethane. To evaluate the validity of our understanding and the generality of the soft-enveloping method, we further applied this method to another replica system.

**Fig. 6:**
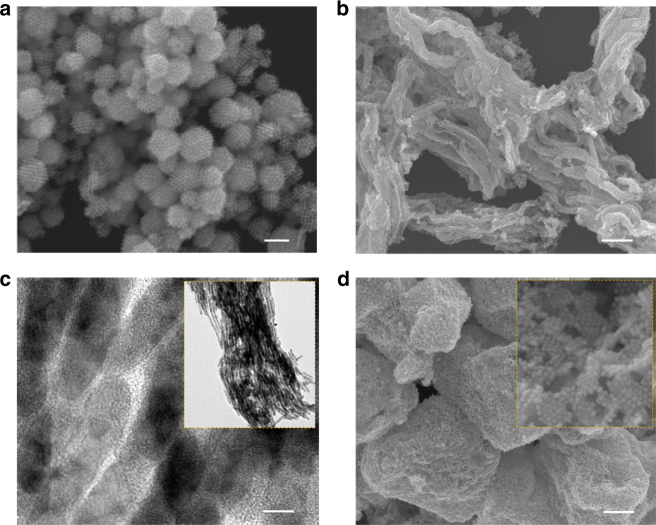
The effectiveness proof of the SLS strategy in other systems. **a** The 3D mesoporous Pt networked structure prepared by using a KIT-6 template. **b**,**c** Ag nanowires obtained via an SBA-15 template. **d** The 3D mesoporous Au nanoparticle superlattice prepared using EP-FDU-12 as the template. The insets in **c** and **d** are the magnified images of corresponding structures obtained from the current soft-enveloped SLS interface reaction strategy. The scale bars in **a**, **b**, **c**, and **d** are 100, 500, 5, and 500 nm, respectively

As shown in Fig. 6, three kinds of Pt, Au and Ag nanostructures are obtained from the current soft-enveloped SLS interface reaction strategy by means of a similar synthetic approach but with changes only to the metal species and templates. Using a KIT-6 mesoporous silica template, the 3D mesoporous Pt networked structure can be prepared (Fig. 6a). Similarly, 1D Ag nanowires, and Au nanoparticle superlattices may also be fabricated using the SBA-15 and EP-FDU-12 mesoporous templates, as shown in Fig. 6b–d. Note that in all the reaction systems (Fig. 6), the products demonstrate a single morphological feature, revealing the deposition of metal nanostructures outside the mesoporous silica template has been effectively avoided. As a comparison, we have also evaluated the validity of the current protocol by removing the solvent barrier layer. It was clearly seen that, when the synthetic system was absent of the barrier layer, bulk NPs can always observed in all three above reaction systems as a result of metal growth on the outer surface of the mesoporous silica template (Supplementary Figs. 28a–c).

## Discussion

In this work, we have proposed a robust synthetic strategy via a general soft-enveloping SLS interface reaction to effectively prevent metal species from migrating to the outside of mesoporous channels. Au or AuAg alloy nanostructures were successfully obtained with diverse morphologies, including spherical NPs, multiple pods, and networks. The 3D mesoporous AuAg networked structure demonstrates outstanding electrocatalytic properties that include enhancements in specific activity and mass activity of 7 and 3 times, respectively, over 4 nm-sized Au NPs. By extending the soft-enveloped SLS interface reaction strategy to other replica systems using different barrier layer solvents, reduction agents, metal species and mesoporous templates, a variety of mesostructures have also been synthesized, including the 3D mesoporous Pt networks, 1D Ag nanowires, and Au nanoparticle superlattices. Finally, our strategy and results offer a synthetic platform to generate a new class of mesoporous metal nanostructures, thus opening avenues to exploit diverse applications in catalysis, optics and biomedicine^55^.

## Methods

### Synthesis of mesoporous Au networks

In a typical impregnation process, the dried KIT-6 (0.1 g) powder was immersed into ethanol solution of HAuCl_4_ (3 mM, 10 ml). Then, the mixed solution was dried under reduced vacuum condition to incorporate the Au precursor into the mesopores. After the complete drying, yellow-colored powder was obtained. Then, the powder was dispersed in hexane (1 ml), TMDS (100 µl) was added into the hexane solution. The Au deposition was carried out in a closed vessel for various periods while the color was changed to black. The obtained power was washed with hexane and ethanol, and dried in room temperature. The silica template was removed by hydrofluoric acid (HF, 20 wt%). Black powder were centrifuged and washed with distilled water and ethanol, then dried up at room temperature.

### Characterization

The morphology and structure of the product were characterized using a scanning electron microscope (SEM, JEOL, JSM-7000F) and a transmission electron microscope (TEM, JEOL, JEM-2100 with an accelerating voltage of 200 kV). The HAADF-STEM image and energy-dispersive spectroscopy elemental mapping of the product were obtained by scanning transmission electron microscopy (STEM, JEOL, JEM-ARM 200F). The chemical composition of the product was characterized using X-ray diffraction (XRD, Bruker), X-ray photoelectron spectroscopy (XPS, Thermo Fisher Scientific, ESCALAB 250Xi+) and Inductive Coupled Plasma Emission Spectrometer (ICP, SHIMADZU, ICPE-9000). The In situ UV spectroscopy measurements were characterized using Ultraviolet–visible spectroscopy (UV, Shimadzu, UV-60).

### Electrochemical measurements

The electrochemical measurement was performed on a VersaSTAT 3 electrochemical working station using a three-electrode cell. The working electrode was a glassy-carbon rotating disk electrode (GCE, 5 mm in diameter). A platinum foil with area of 1 cm^2^ was used as the counter electrode, and a double junction Ag/AgCl electrode was used as the reference. Before the measurement for the catalytic property, the catalyst was firstly treated by 30 cycles of CV (between 0.3 and 0.7 V) in 0.5 M KOH aqueous solution with a scan rate of 50 mV s^−1^ to remove the remaining of SiO_2._ The samples for the measurement of Au nanoparticles were not treated with KOH solutions. Then, the measurement for the catalytic property was performed in deoxygenated solution of 0.5 M KOH and 2 M methanol with a scan rate of 10 mV s^−1^. The active surface area of various catalysts was estimated using the CV curve measured in deoxygenated 0.5 M H_2_SO_4_ aqueous solution at a scan rate of 10 mV s^−1^. The accelerated durability test (ADT) was performed using CV cycles in O_2_ saturated 0.5 M KOH + 2 M CH_3_OH aqueous solution with scanningfrom −0.15 to 0.55 V (vs Ag/AgCl) at rate of 10 mV s^−1^. In order to avoid the effect of the decrease of methanol concentration on the results, very 500 cycles, new prepared solution is used to complete the ADT.

### Data availability

The authors declare that all the data supporting the findings of this study are available within the paper and its Supplementary Information or from the corresponding author upon reasonable request.

## Electronic supplementary material

Supplementary Information
