# Supplementary material for: A general soft-enveloping strategy in the templating synthesis of mesoporous metal nanostructures
Source: Nat Commun. 2018 Feb 6;9:521. doi: 10.1038/s41467-018-02930-9 (PMC5802826; doi:10.1038/s41467-018-02930-9)
Supplement: Supplementary file 1 — Supplementary Information [file 41467_2018_2930_MOESM1_ESM.pdf]

Supplementary Information

A general soft-enveloping strategy in the templating synthesis of mesoporous metal nanostructures

Fang et al.

Supplementary Methods

Materials. KIT-6 was purchased from Nanjing Material Tech. Co., Ltd.; chloroauricacid ($HAuCl_4$•$4H_2O$), potassium tetrachloroplatinate (K_2PtCl_4), hexane (C_6H_{14}), hydrofluoric acid (HF), sodium hydroxide (NaOH) and anhydrous ethanol (C_2H_5OH) were obtained from Sinopharm Chemical Industry Co., Ltd.; Silver nitrate ($AgNO_3$) and 1,1,3,3-tetramethyldisiloxane (TMDS) were purchased from Shanghai Aladdin Biochemical Technology Co., Ltd.. All chemicals were used without further purification. Milli-Q water (>18.0 MΩ cm) was purified with a Sartorius arium611 UV ultrapure water system.

Synthesis of mesoporous Au networks. KIT-6 was dried under reduced pressure at 120℃ to remove adsorbed water. In a typical impregnation process, the dried KIT-6 (0.1 g) powder was immersed into ethanol solution of $HAuCl_4$ (3 mM, 10 ml). Then, the mixed solution was dried under reduced vacuum condition to incorporate the Au precursor into the mesopores. After the complete drying, yellow-colored powder was obtained. Then, the powder was dispersed in hexane (1 ml), TMDS (100 ul) was added to the hexane solution. The Au deposition was carried out in a closed vessel for 1 h while the color was changed to black. The obtained power was washed with hexane and ethanol, and dried in room temperature. The silica template was removed by hydrofluoric acid (HF, 20 wt%). Black powder were centrifuged and washed with distilled water and ethanol, then dried up at room temperature.

Synthesis of mesoporous Au-Ag networks. The only difference for the synthesis of the Au-Ag networks was as follows: After KIT-6 was dried, Ag was loaded on mesopores by impregnating $AgNO_3$ with different mole percent to Au (4%, 8%, and 12%) dissolved in the mixture of mesopores KIT-6 and ethanol. All other processes were same.

Synthesis of the Au nanoparticles. A) 3 nm, 4 nm and 5 nm NPs: A 15 ml of freshly prepared reducing solution of sodium citrate (SC, 2.2 mM) containing 0.1 ml of tannic acid (TA, 2.5 mM)

was heated under vigorous stirring. When the temperature reached 70 ℃, 0.1 ml of tetrachloroauric acid ($HAuCl_4$, 25 mM) was injected. The solution was kept at 70℃ for more 5 minutes to obtain gold precursor particles (3-nm Au nanoparticles). For further growing the Au nanoparticles, the solution was diluted (1:3) by extracting 5.5 ml of the sample and adding 5.5 ml of SC (2.2 mM). When the temperature reached 70 ℃ again, 0.05 ml of $HAuCl_4$ (25 mM) were added. After 5 minutes, 4-nm Au nanoparticles were prepared. By subsequently repeating this process, dilution plus two injections, 5-nm Au nanoparticles were grown. B) 8 nm: In a typical procedure, a 10 ml $HAuCl_4$ (0.25 mM) was put into a three-neck flat-bottom flask. After the solution was brought to a boil under reflux with stirring, 5 ml of 1 % sodium citrate solution was added. The reaction solution was allowed to boil until the solution became wine red in color (about 20 min). The gold nanoparticles were kept at 4 ℃. C) 70-nm: An amount of 10 ml of the as-synthesized 8 nm seed particles was added to a 1 ml growth solution containing 55 mM CTAB, 25 mM $HAuCl_4$ and 4 mM ascorbic acid. The solution was kept undisturbed for 1 h at room temperature and allowed to cool to room temperature.

Synthesis of mesoporous Pt networks. In the Pt deposition process, KIT-6 (0.1 g) powder was immersed into K_2PtCl_4 (0.1 M, 10 ml) aqueous solution and the composite was dried under reduced vacuum condition. Then, the powder was reduced with hexane and TMDS in the same way. After that, all the processes were same.

Synthesis of Ag nanowires. In the Ag deposition process, SBA-15 (0.1 g) powder was immersed into $AgNO_3$ (0.8 M, 0.125 ml) aqueous solution in stirring for 30 minutes and the composite was dried under reduced vacuum condition. Then, the power was reduced with hexane and TMDS in the same way. The silica template was removal by NaOH (5 M, 100 ml) in stirring and washed with distilled water and ethanol.

Synthesis of Au nanoparticle superlattice. In the Au deposition process, FDU-12 (0.1 g) powder

was immersed into $HAuCl_4$ (3 M, 0.1 ml) aqueous solution in stirring for 30 minutes and the composite was dried under reduced vacuum condition. Then, the power was reduced with hexane and TMDS in the same way. The silica template was removed by NaOH (5 M, 100 ml) in stirring and washed with distilled water and ethanol.

Characterization. The morphology and structure of the product were characterized using a scanning electron microscope (SEM, JEOL, JSM-7000F) and a transmission electron microscope (TEM, JEOL, JEM-2100 with an accelerating voltage of 200 kV). The HAADF-STEM image and energy-dispersive spectroscopy elemental mapping of the product were obtained by scanning transmission electron microscopy (STEM, JEOL, JEM-ARM 200F). The chemical composition of the product was characterized using X-ray diffraction (XRD， Bruker， d8advance), X-ray photoelectron spectroscopy (XPS, Thermo Fisher Scientific, ESCALAB 250Xi+) and Inductive Coupled Plasma Emission Spectrometer (ICP, SHIMADZU, ICPE-9000). The In situ UV spectroscopy measurements were characterized using Ultraviolet–visible spectroscopy (UV, SHIMADZU, UV-60).

Mesoporous Au or Au-Ag networked NP modified electrode preparation. A quantity of 2 mg of mesoporous AuAg networked structure was suspended in 4 ml DI-water by ultrasonication. A quantity of 12 μl of the solution was drop-casted on the glassy-carbon rotating disk electrode surface. The electrode was dried at room temperature under ambient conditions.

Electrochemical measurements. The electrochemical measurement was performed on a VersaSTAT 3 electrochemical working station using a three-electrode cell. The working electrode was a glassy-carbon rotating disk electrode (GCE, 5 mm in diameter). A platinum foil with area of 1 cm^2 was used as the counter electrode, and a double junction Ag/AgCl electrode was used as the reference. Before the measurement for the catalytic property, the catalyst was firstly treated by 30 cycles of CV (between 0.3 to 0.7 V) in 0.5 M KOH aqueous solution with a scan rate of 50 mV s^{-1}

to remove the remaining of SiO_2. The samples for the measurement of Au nanoparticles were not treated with KOH solutions. Then, the measurement for the catalytic property was performed in deoxygenated solution of 0.5 M KOH and 2 M methanol with a scan rate of 10 mV s^{-1}. The active surface area of various catalysts was estimated using the CV curve measured in deoxygenated 0.5 M H_2SO_4 aqueous solution at a scan rate of 10 mV s^{-1}. The accelerated durability test (ADT) was performed using CV cycles in O_2 saturated 0.5 M KOH + 2 M CH_3OH aqueous solution with scanningfrom -0.15 to 0.55 V (vs Ag/AgCl) at rate of 10 mV s^{-1}. In order to avoid the effect of the decrease of methanol concentration on the results, very 500 cycles, new prepared solution is used to complete the ADT.

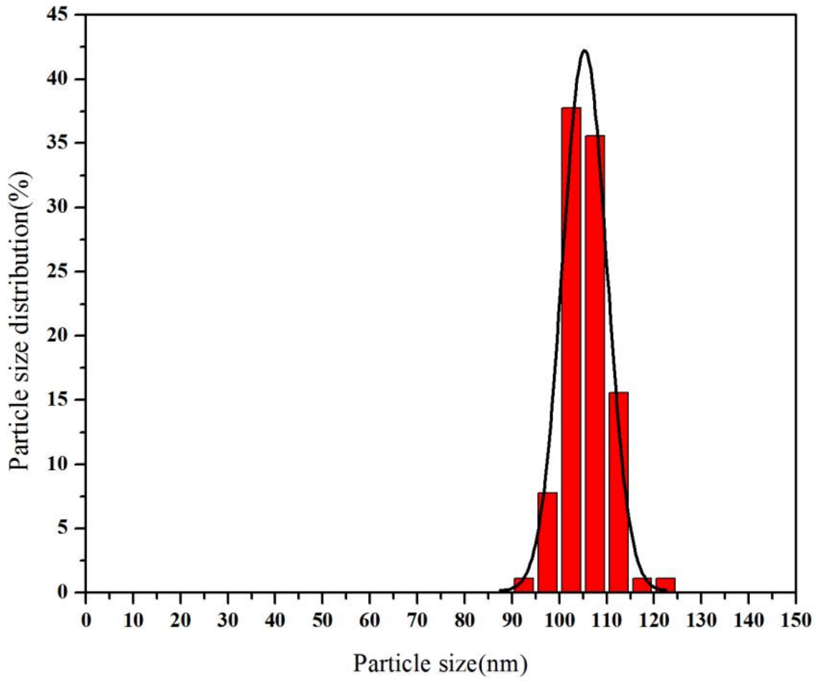

Supplementary Figure 1. Size distribution of 3D Au mesoporous networks shown in Figure 2a.

The average diameter is of ~105 nm, and standard deviation of ~ 4.66.

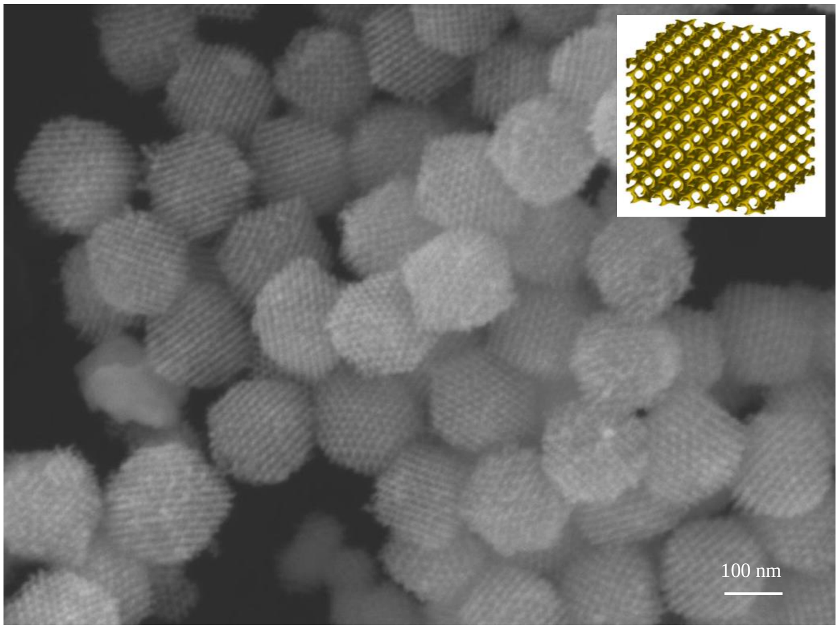

Supplementary Figure 2. SEM and 3D nanostructures (inset) of the typical 3D mesoporous AuAg networks after the removal of the silica template. The profile displays a polyhedral morphology, probably cubic shape.

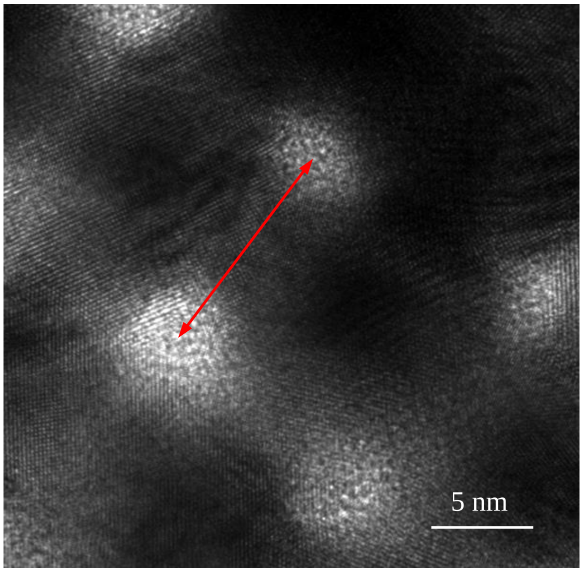

Supplementary Figure 3. Large-sized mesopores of around 14~15 nm were created, corresponding to the total value of the wall thickness plus the pore size of KIT-6. This square-shaped network structure indicates that the mesoporous Au-Ag NPs replicated one side pore system of the bicontinous structure.

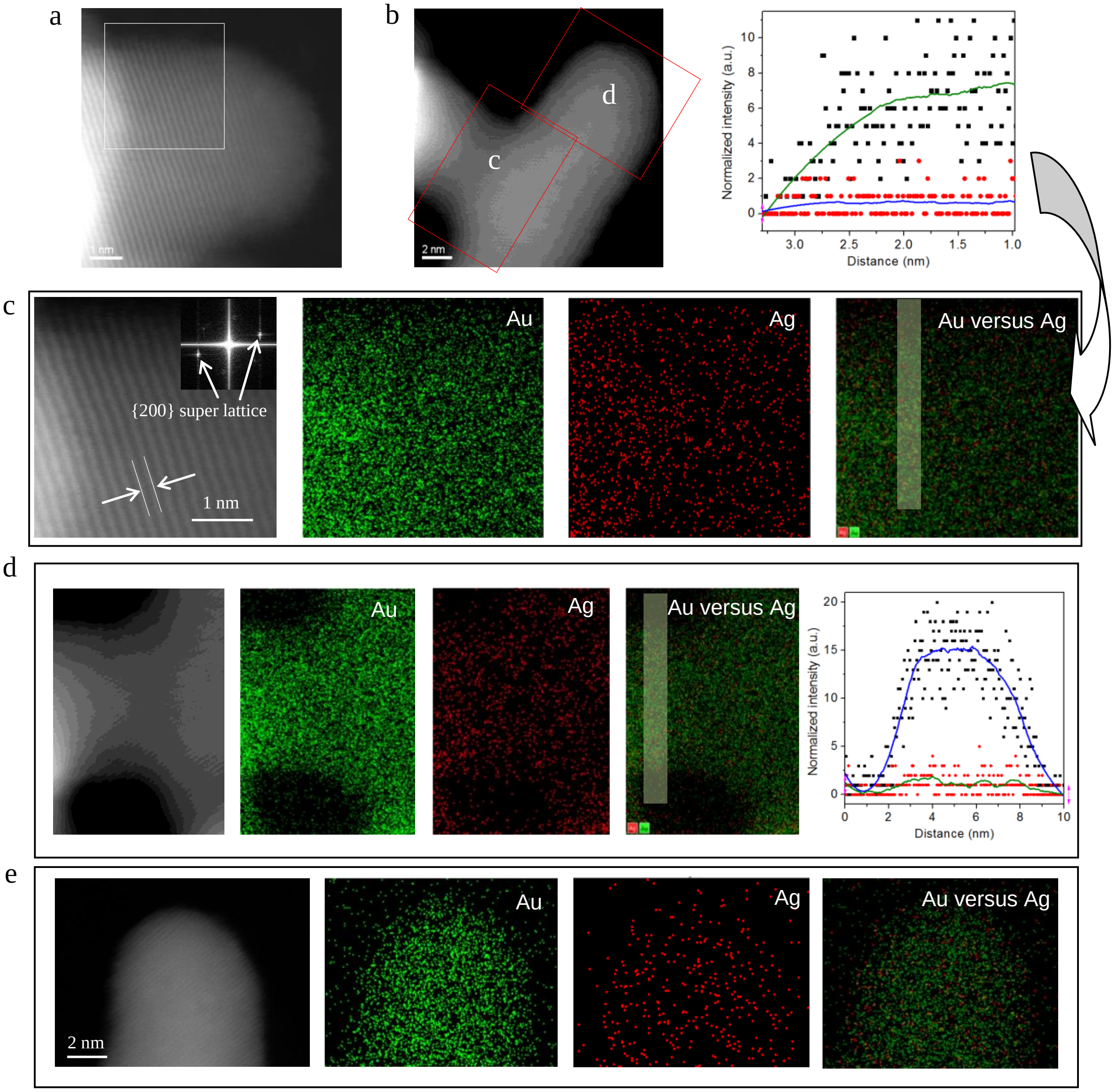

Supplementary Figure 4. (a-e) Representative aberration-corrected, high-angle annular dark-field scanning transmission electron microscopy (HAADF- STEM) characterizations in combination with energy dispersive X-ray spectroscopy (EDX) at different regions and different magnified resolutions from atomic level to nanometer scale.

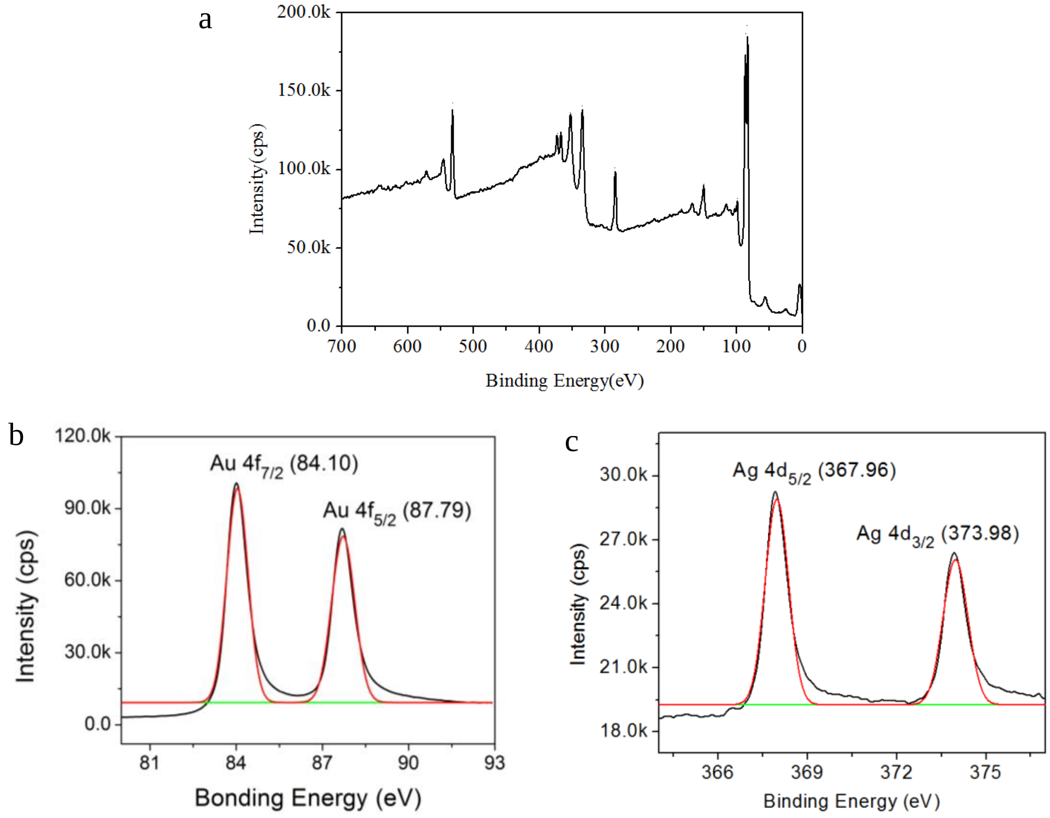

Supplementary Figure 5. (a) XPS spectra of mesoporous Au-Ag networked NPs. (b) Au 4f and (c) Ag 4d XPS spectra. Compared with the standard binding energies of Au $4f_{7/2}$ (84.0 eV) and Au $4f_{5/2}$ (87.70 eV), these two peaks of Au-Ag networked NPs are shifted to higher energy a little (around 0.1 eV), with values of 84.10 eV and 87.79 eV, respectively. On the other hand, compared with the standard data, the peaks of Ag $4d_{5/2}$ and Ag $4d_{3/2}$ are shifted conversely to lower energy, that are 367.96 eV and 373.98 eV, respectively. The ratio of Ag atoms in AuAg networked NPs is around 6.9%, so most of the Ag atoms are dispersed in the AuAg alloy and contacted with Au atoms. While, the ratio of Au atoms is around 93.1%, so most of Au atoms are contacted each other. Thus, the peaks of Ag $4d_{5/2}$ and Ag $4d_{3/2}$ show an obvious shift and the peaks of Au $4f_{7/2}$ and Au $4f_{5/2}$ show a little shift.

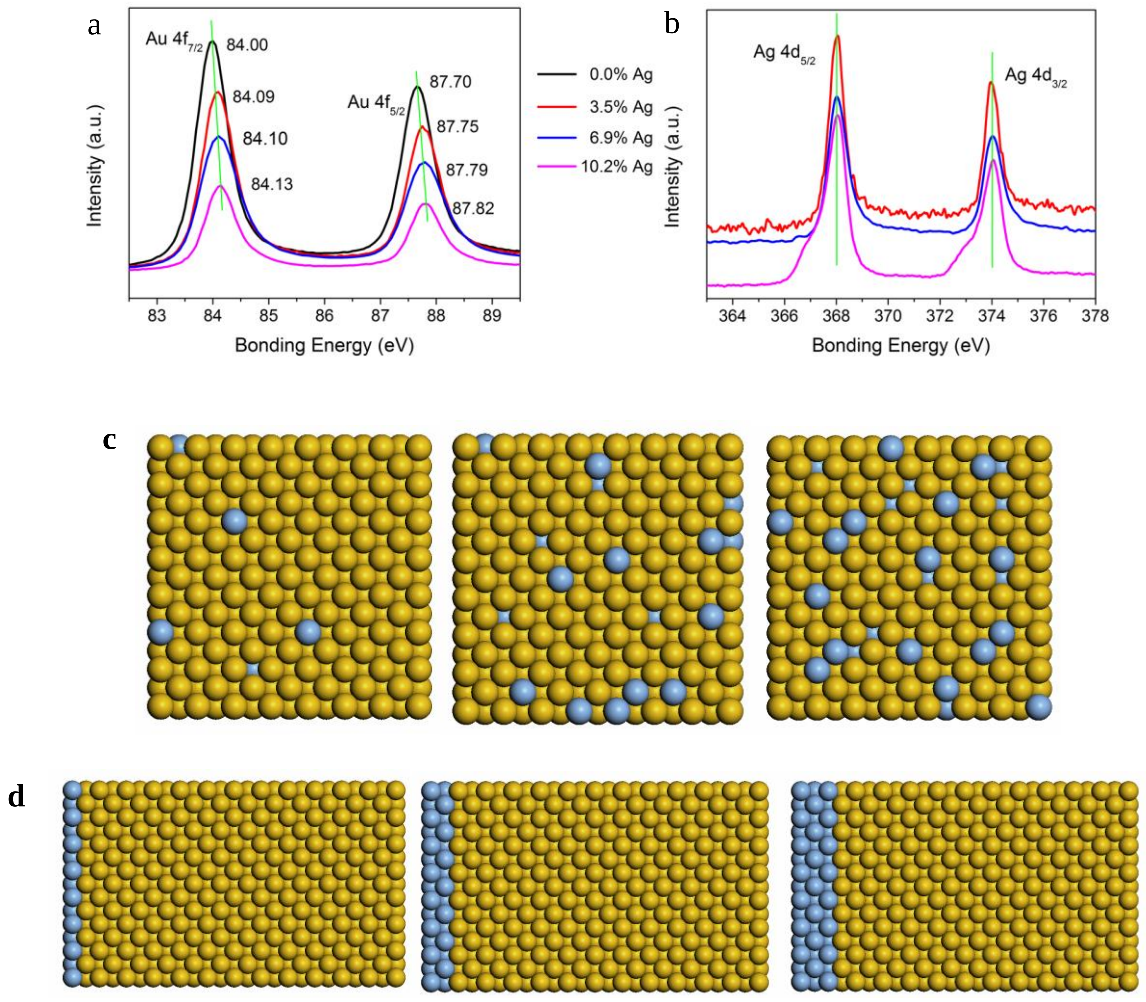

Supplementary Figure 6. (a) Au 4f and (b) Ag 4d XPS spectra of mesoporous AuAg networked NPs with the content of Ag changing from 0% to 12%.With the increasing of Ag, the peaks of Au $4f_{7/2}$ and Au $4f_{5/2}$ shifted to higher bonding energy gradually. While, the peaks of Ag $4d_{5/2}$ and Ag $4d_{3/2}$ keep no obvious change, as the content of Ag change from 3.5% to 12%. **(c)-(d),** Schematic images of atomic distribution in (c) AuAg alloy and (d) AuAg unalloyed nanocrystals with Ag content increase from 3.5% to 6.9% and 12%. As shown in Supplementary Figure 6c, Ag atomic ratio in AuAg alloy is relatively much lower than Au atomic ratio. The most of Ag atoms are dispersed in the Au matrix and most of adjacent atoms for Ag are Au atoms. Thus, the XPS peaks of Ag $4d_{5/2}$ and Ag $4d_{3/2}$ keep no obvious change, as the content of Ag change from 3.5% to 12%. On the contrary, with the Ag content increase, the number of Au atoms neighboring with Ag atoms obviously increase. Thus, the XPS peaks of Au $4f_{7/2}$ and Au $4f_{5/2}$ shifted to higher bonding energy

gradually. As shown in Supplementary Figure 6d, in the AuAg unalloy crystal, Ag atoms connect with Ag atoms and Au atoms connect with Au atoms. Thus, even the ratio of Ag atoms increases, the peaks for Au and Ag would have no obvious shifting on the XPS spectra.

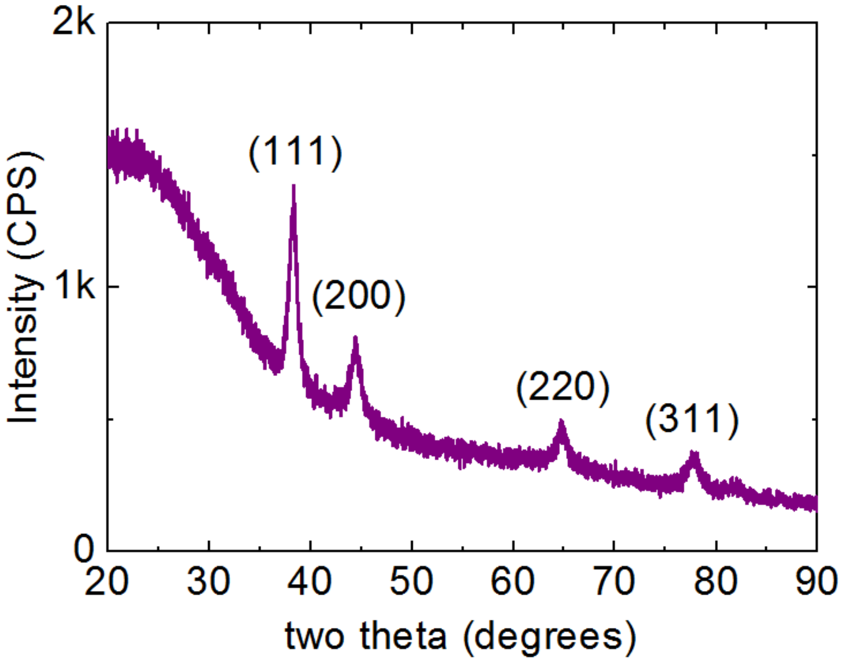

Supplementary Figure 7. XRD curve of mesoporous Au-Ag networked NPs.

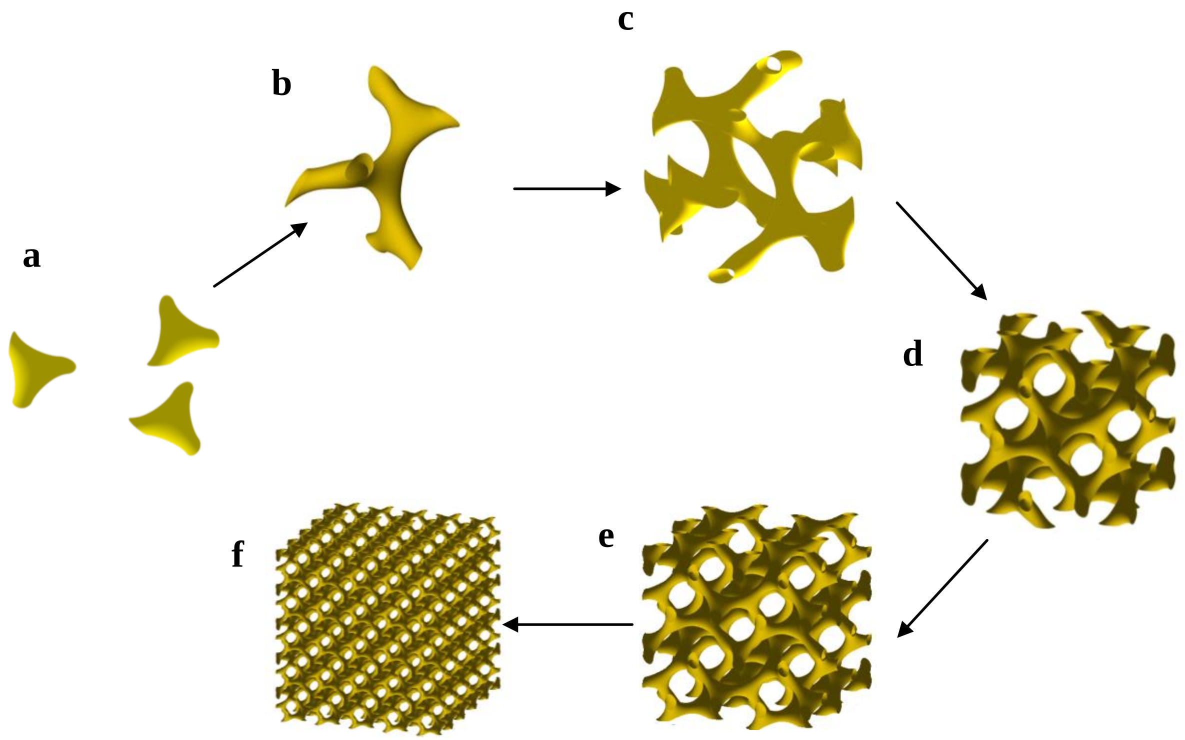

Supplementary Figure 8. (a-f) Schematic structures of nanostructured Au obtained from the encapsulated nanocasting process using KIT-6 mesoporous silica as the template and the chemical reduction at different stages.

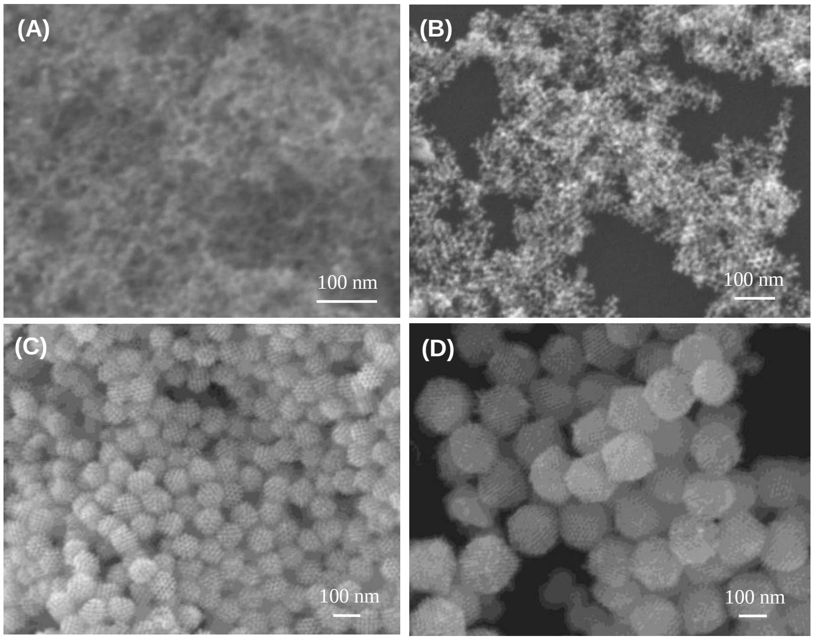

Supplementary Figure 9. SEM images of nanostructured Au obtained from the encapsulated nanocasting process using KIT-6 mesoporous silica as the template and a chemical reduction at 3 mM $HAuCl_4$ concentration and at different stages: a) Multiple-pod Au nanostructure from the growth time of 10 min, b) - d) mesoporous Au NPs obtained after 30 min (b), 6 h (c), and 24 h (d) reaction time.

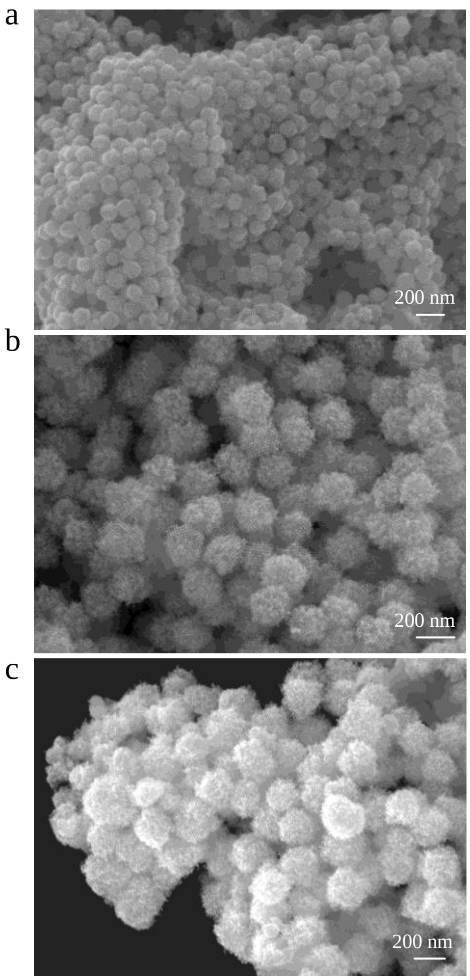

Supplementary Figure 10. 3D mesoporous Au networked NPs synthesized at relatively low or high $HAuCl_4$ concentrations: a, 0.5 mM, b, 10 mM and c, 100 mM.

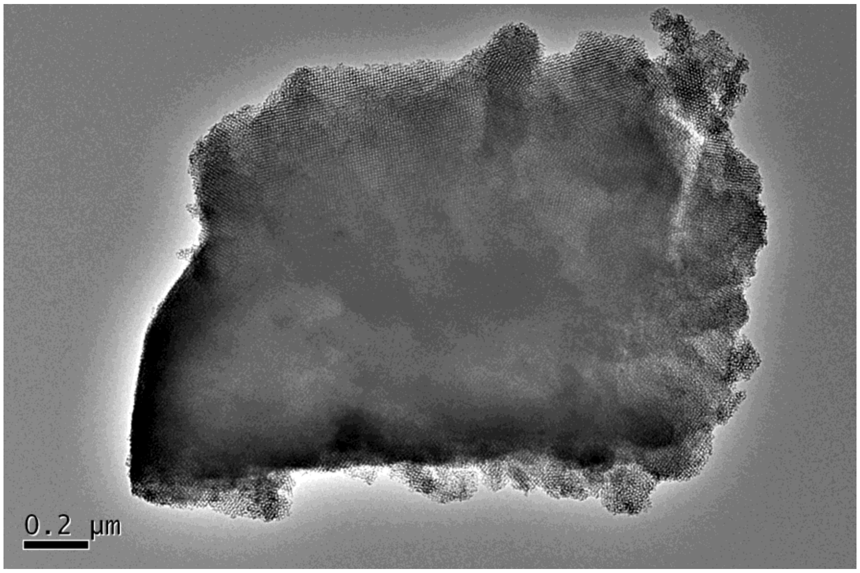

Supplementary Figure 11. TEM image of the Au precursor/KIT-6 composites, in which the Au precursor distributed homogeneously in the ordered mesoporous silica matrix.

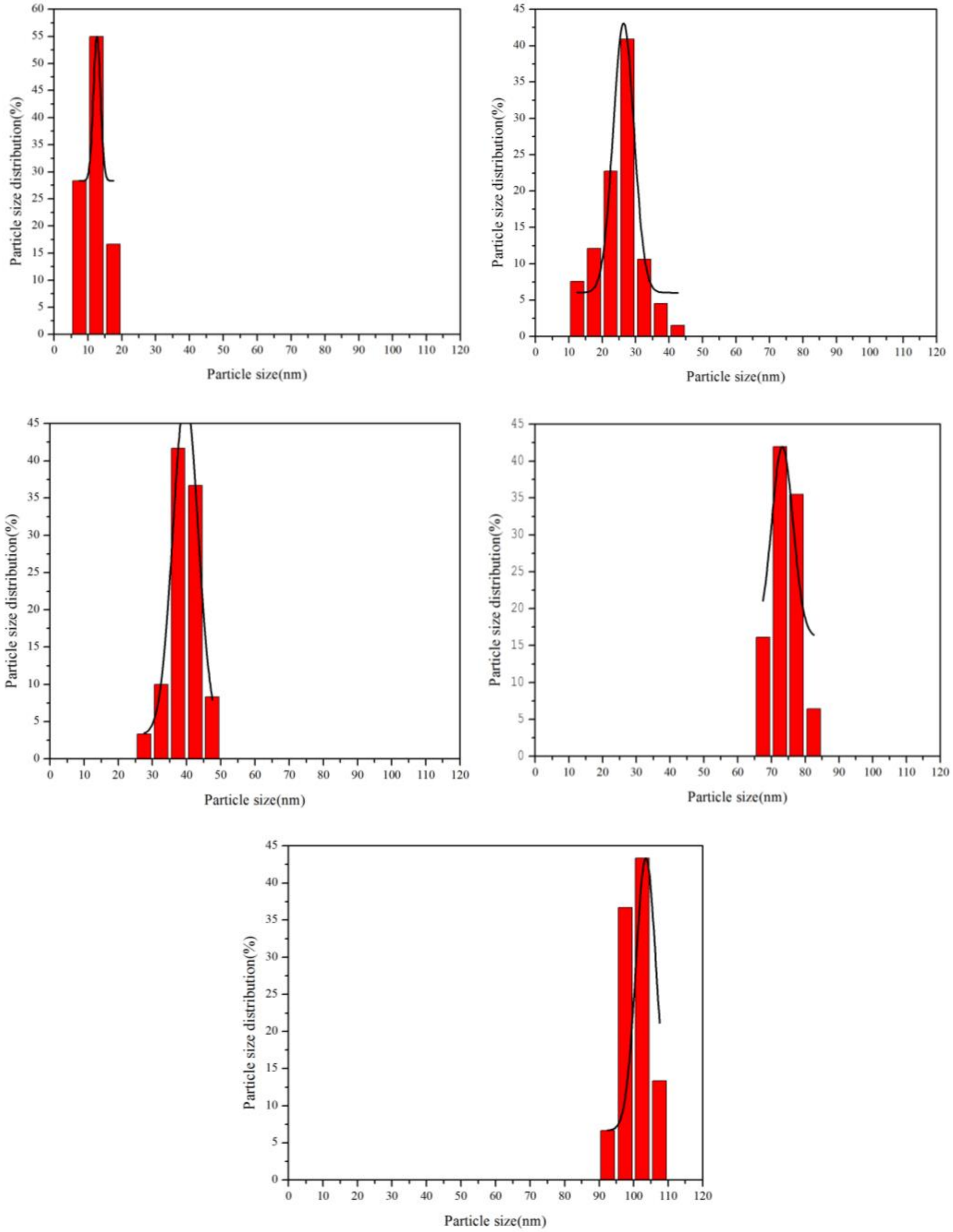

Supplementary Figure 12. The size distributions of Au nanostructures shown in Figures 3b-f.

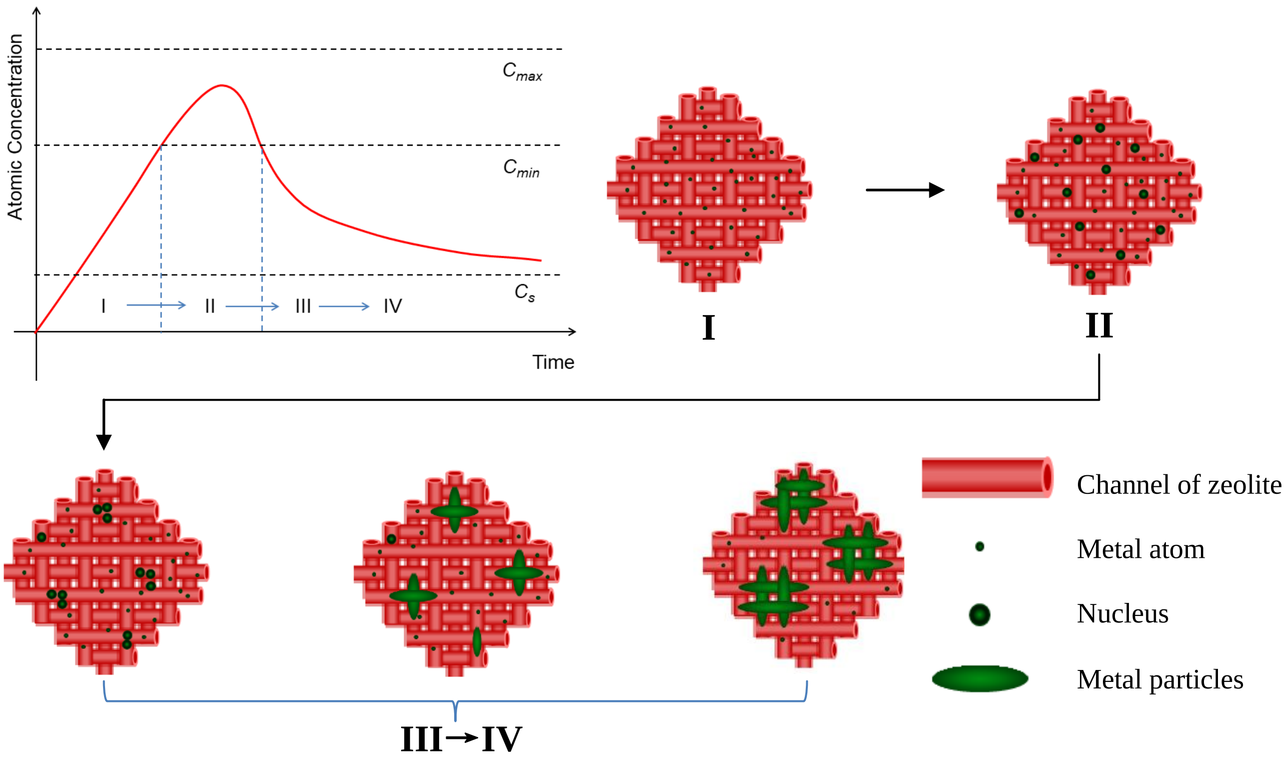

Supplementary Figure 13. The proposed growth mechanism of 3D mesoporous Au networks, which can be described by the classical LaMer curve and seems to consist of three distinct periods, i.e. (i) an induction period; (ii) nucleation period; (iii-iv) The growth period of Au multiple-pods and mesoporous network.

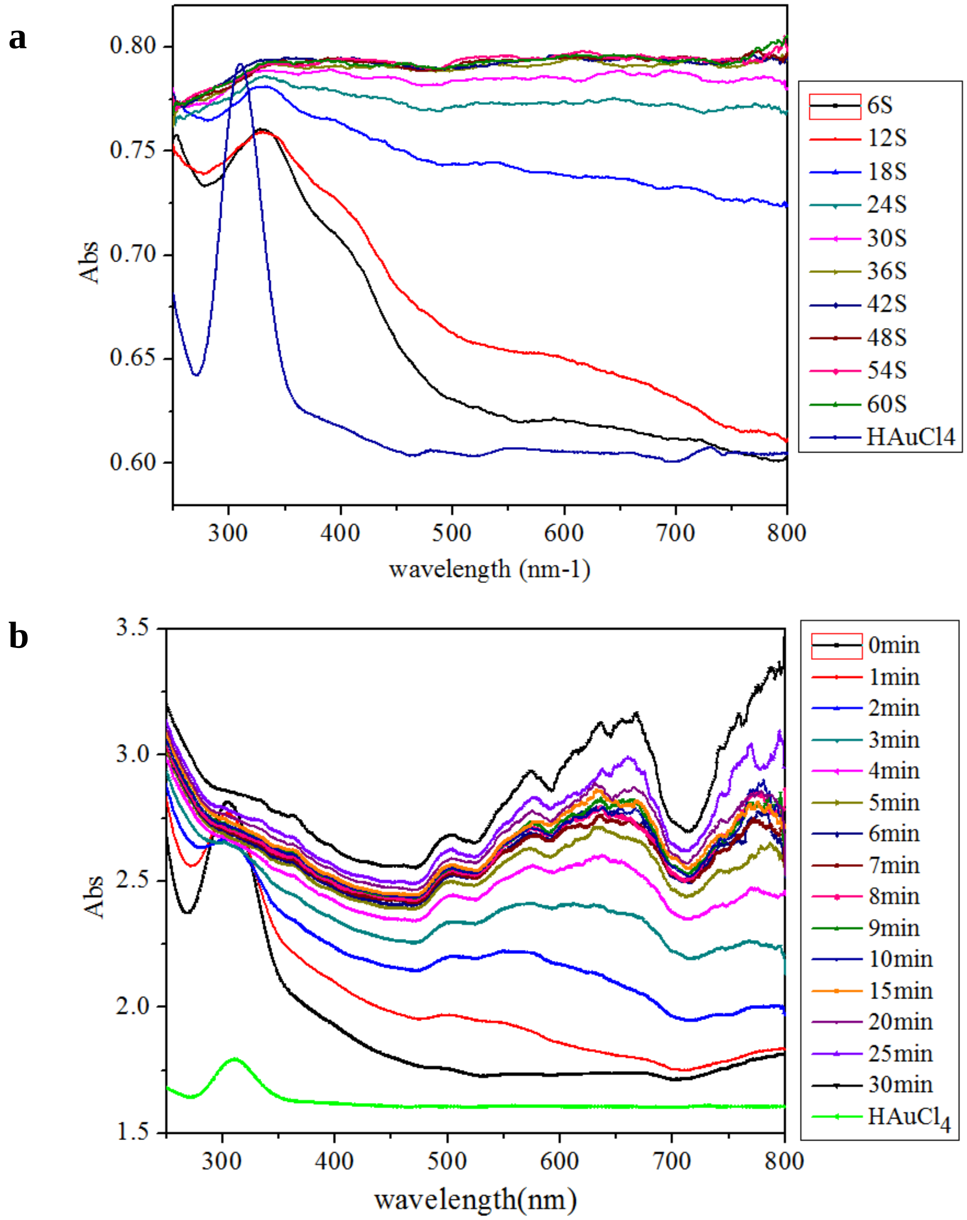

Supplementary Figure 14. In situ UV spectroscopy measurements of growth for 3D mesoporous Au networked nanostructures. a, within ~60 s, b, ~30 min. The early growth stage shows Au cluster peaks at around 400 nm, and these peaks did not appear again in the following growth period.

a

b

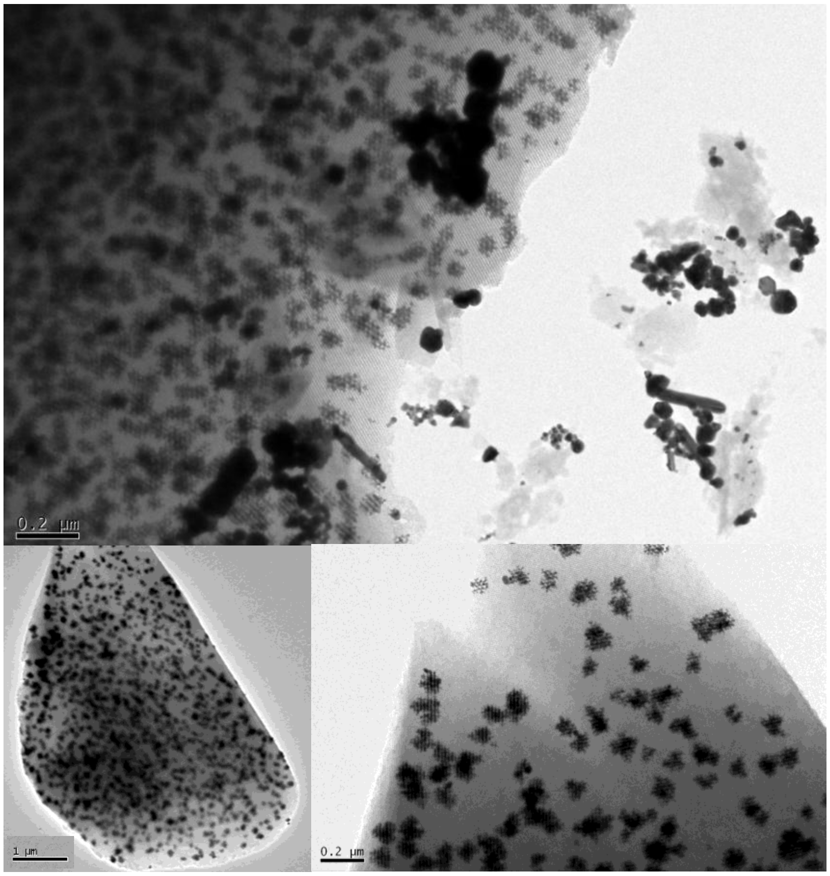

Supplementary Figure 15. a, When the synthetic system was absent of the barrier layer ('liquid'), some particles without ordered mesoporous structure can be obviously observed as a result of Au growth on the outer surface of the mesoporous silica template. **b**, With current encapsulated SLS interface reaction strategy, the Au species can be effectively controlled to grow inside mesoporous silica.

a

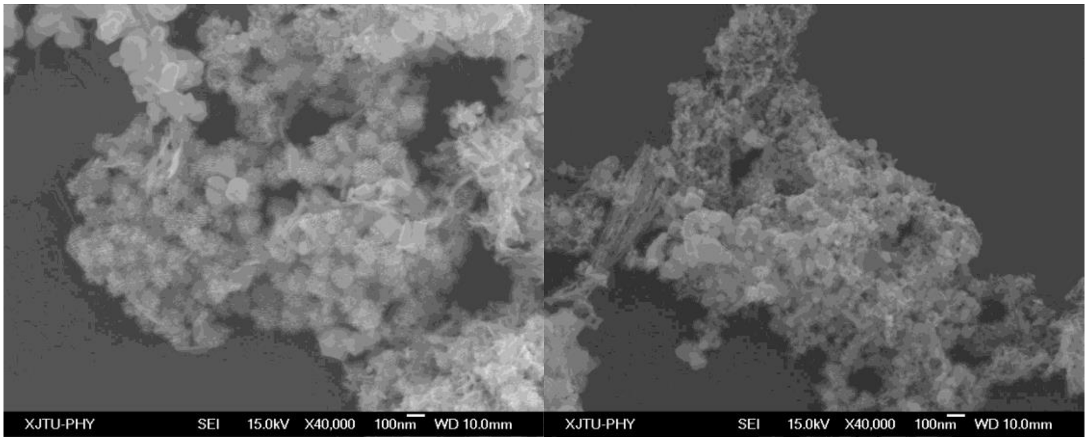

b

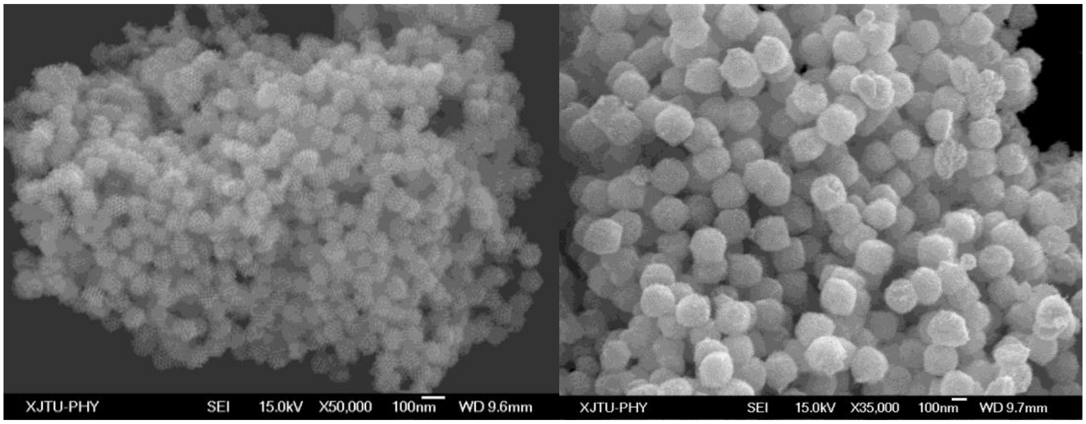

Supplementary Figure 16. a, When the synthetic system was absent of the barrier layer ('liquid'), some particles without ordered mesoporous structure can be obviously observed as a result of Au growth on the outer surface of the mesoporous silica template. **b**, With current encapsulated SLS interface reaction strategy, the Au species can be effectively controlled to grow inside mesoporous silica.

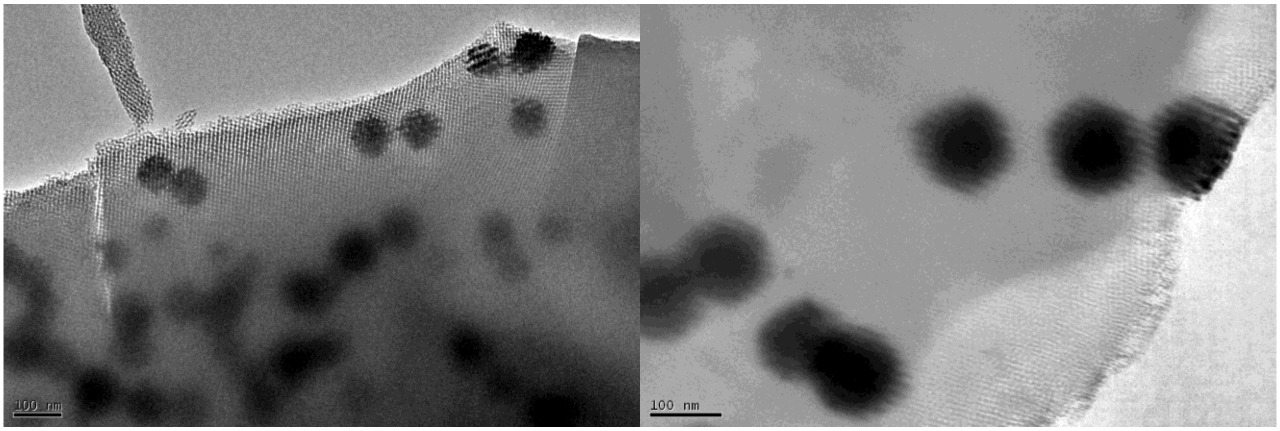

Supplementary Figure 17. 3D Au mesoporous networked structures within KIT-6 template reduction by dimethylaminoborane (DMAB)

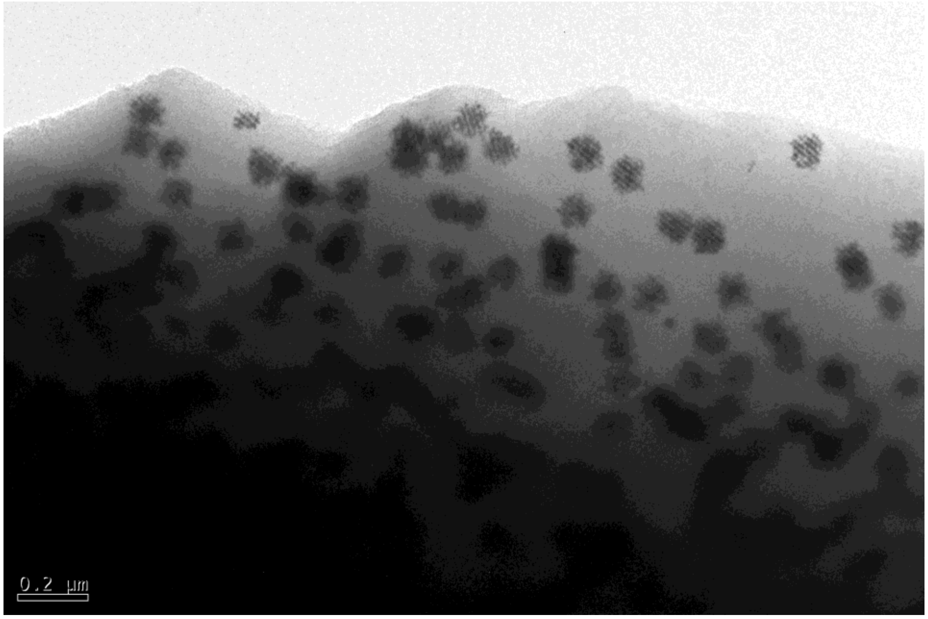

Supplementary Figure 18. 3D Au mesoporous networked structures within KIT-6 template reduction by butylamine.

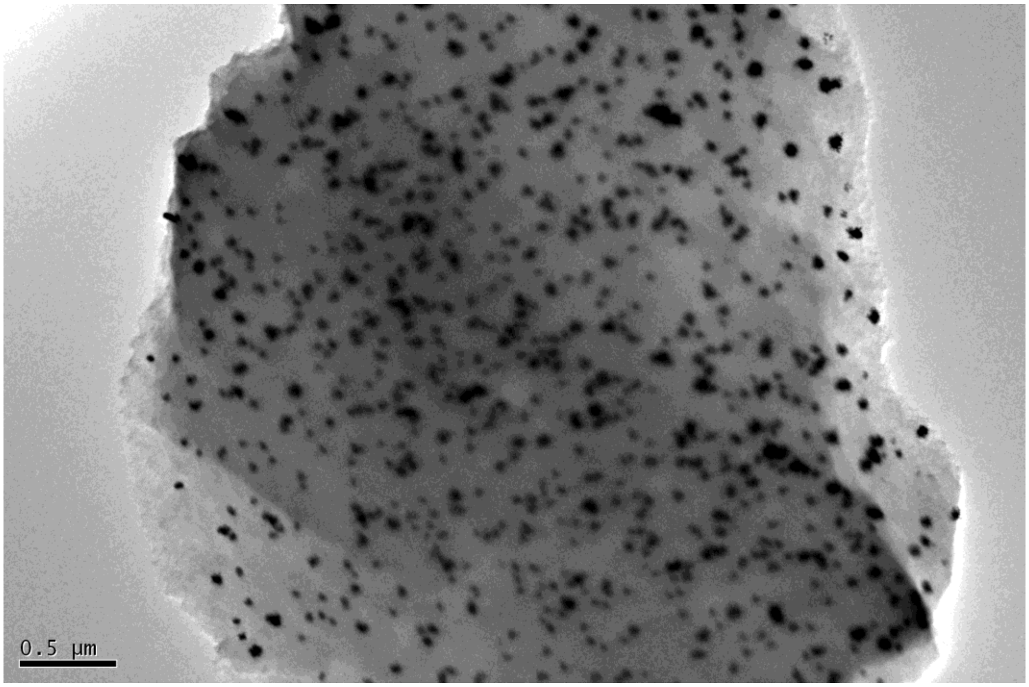

Supplementary Figure 19. 3D Au mesoporous networked structures within KIT-6 template reduction by TMDS, but with dichloromethane as soft-enveloping solvent layer.

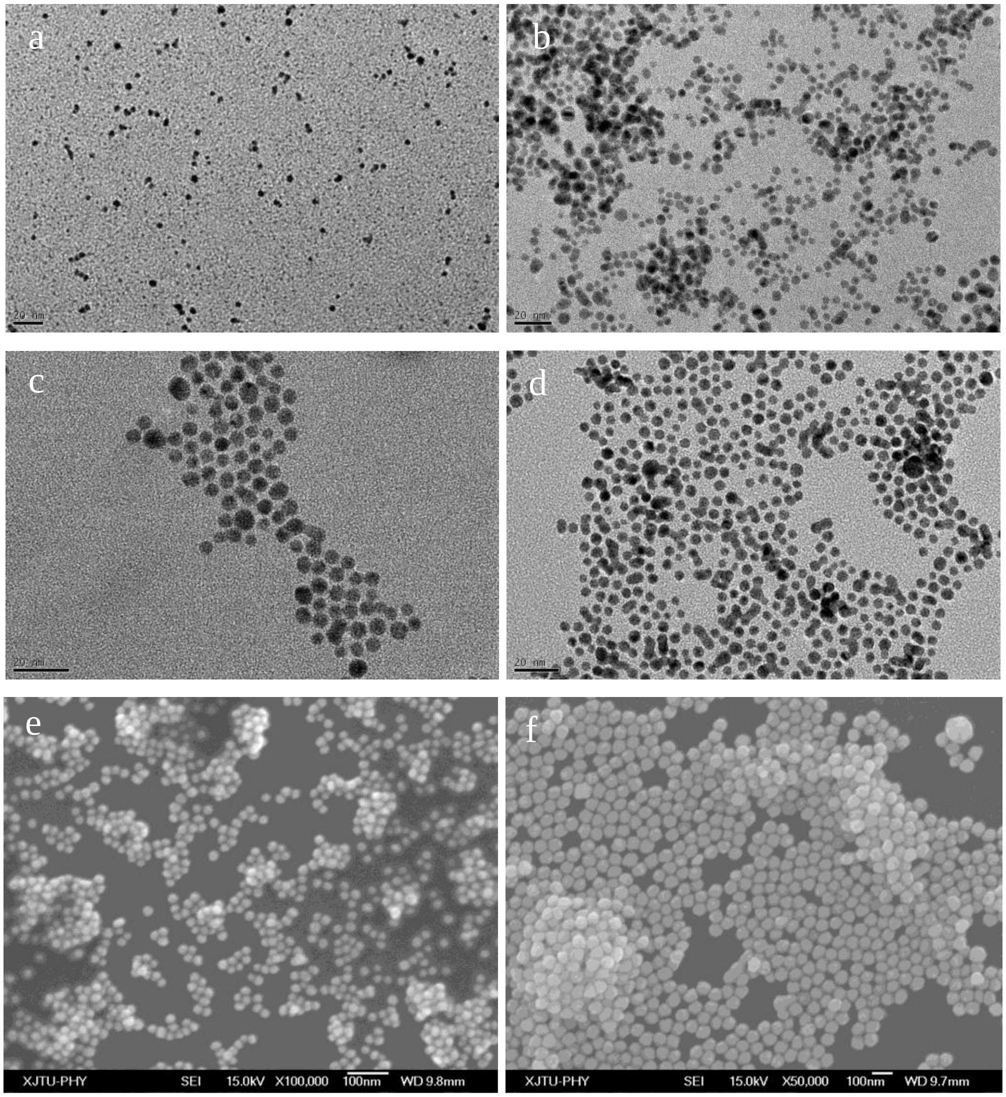

Supplementary Figure 20. TEM or SEM images of Au nanoparticles with size of (a) 3 nm, (b) 4 nm, (c) 5 nm, (d) 6 nm, (e) 20 nm and (f) 70 nm.

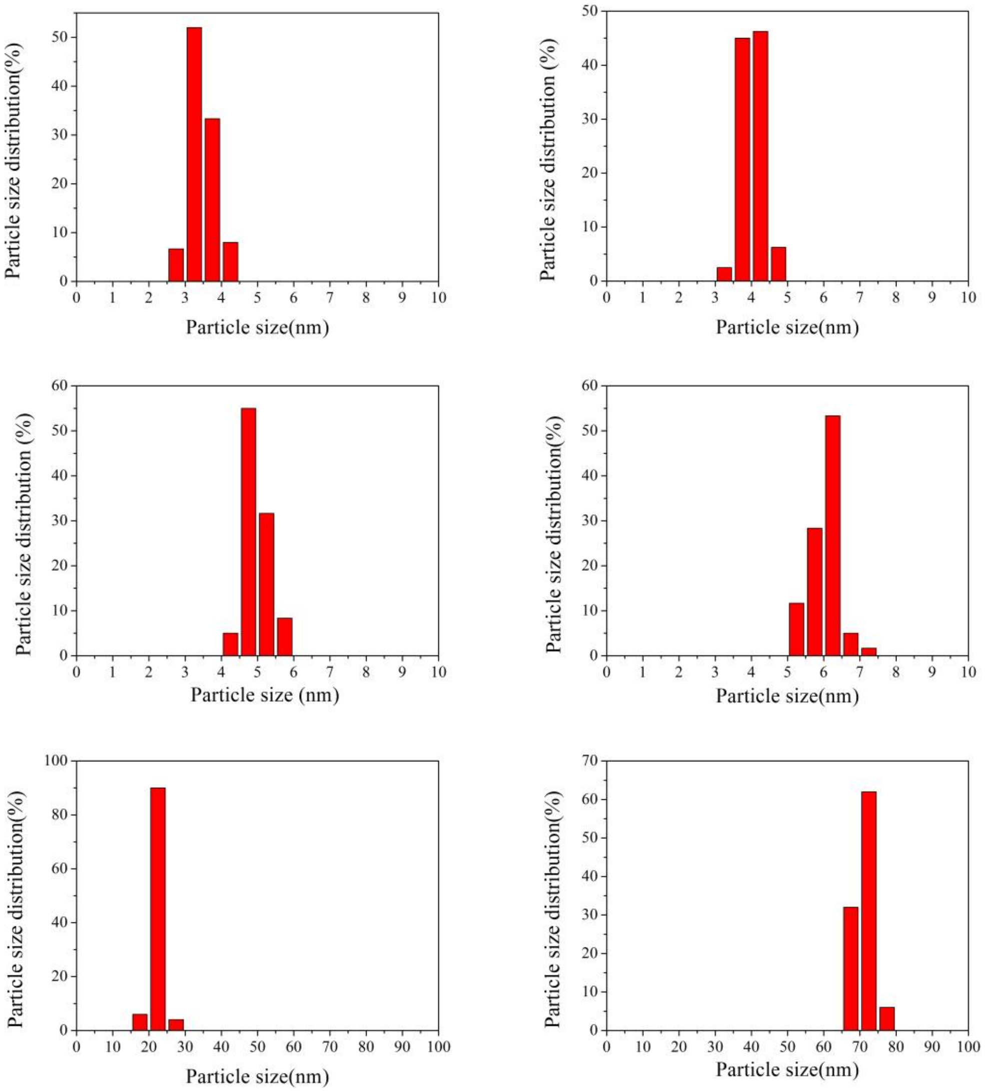

Supplementary Figure 21. The size distributions of Au nanoparticles shown in Figure 20.

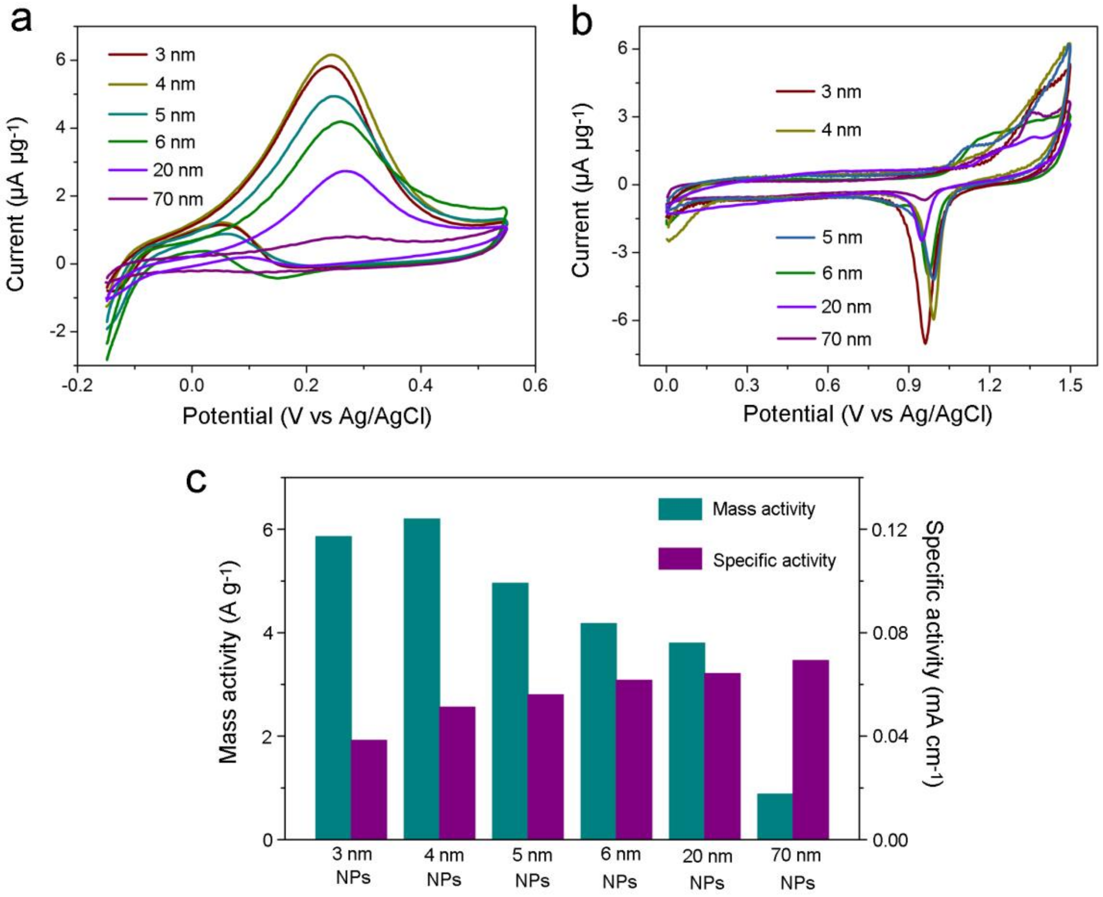

Supplementary Figure 22. Electrochemical performance of Au nanoparticles (NPs) with different size: 3 nm, 4 nm, 5 nm, 6 nm, 20 nm, and 70nm. a) Cyclic voltammograms of electro-methanol oxidation, b) oxide stripping curves, c) histograms of mass activity and specific activity. The cyclic voltammograms were performed in 0.5 M KOH and 2 M CH_3OH at scan rate of 10 mV s^{-1}. The oxide stripping was measured in 0.5 M H_2SO_4 solution at a scan rate of 10 mV s^{-1}.

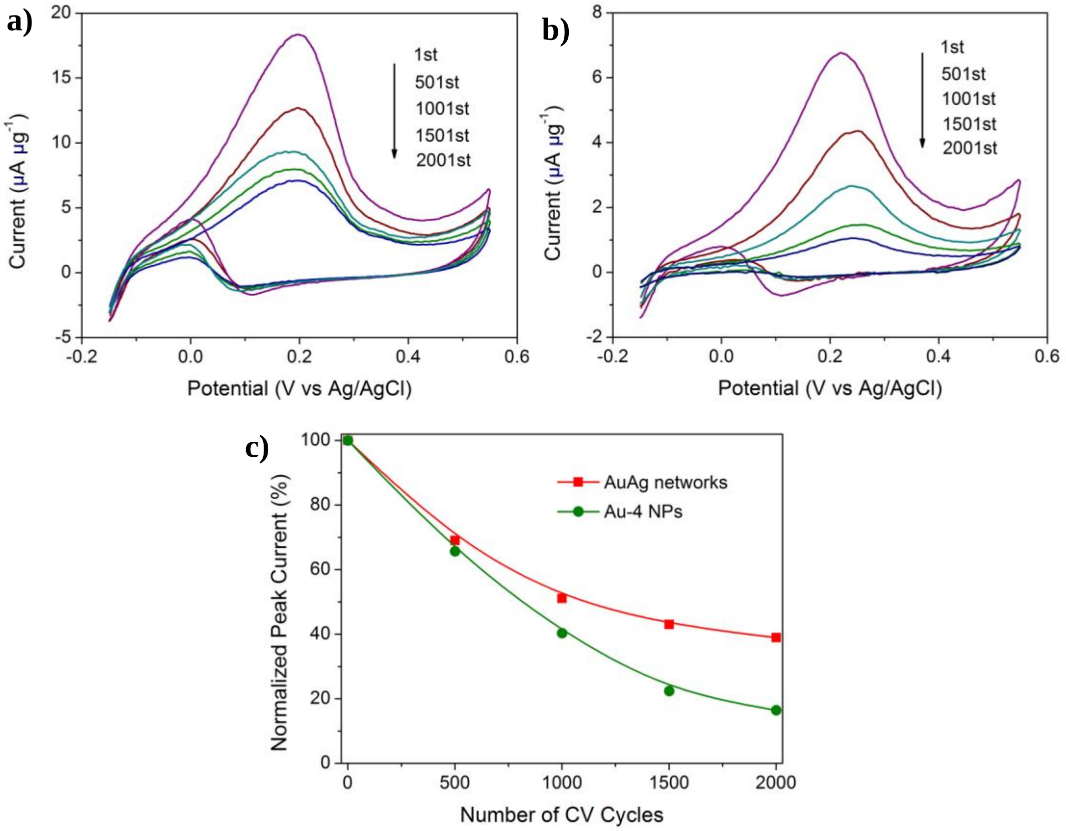

Supplementary Figure 23. Comparison of electrochemical durability for the AuAg networks and Au-4 NPs.CV curves obtained during the accelerated durability test (ADT) for a) AuAg networks and b) Au-4 NPs after different cycles. The ADT was performed in O_2 saturated 0.5 M KOH + 2 M CH_3OH aqueous solution with scanningfrom -0.15 to 0.55 V (vs Ag/AgCl) at rate of 10 mV s^{-1}. c) The loss of normalized peak currents for AuAg networks and Au-4 NPswith the increase of CV cycles.

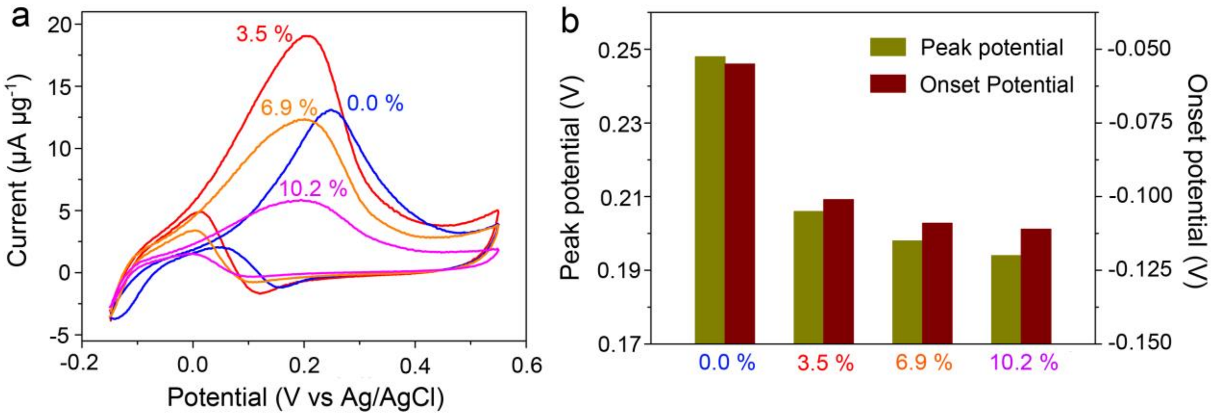

Supplementary Figure 24. Composition-dependent electrochemical performance of AuAg networked NPs. a) Cyclic voltammograms of electro-methanol oxidation, and d) histograms of peak potential and onset potential of the methanol oxidation of AuAg networked NPs with Ag composition changing from 0 to 12%.

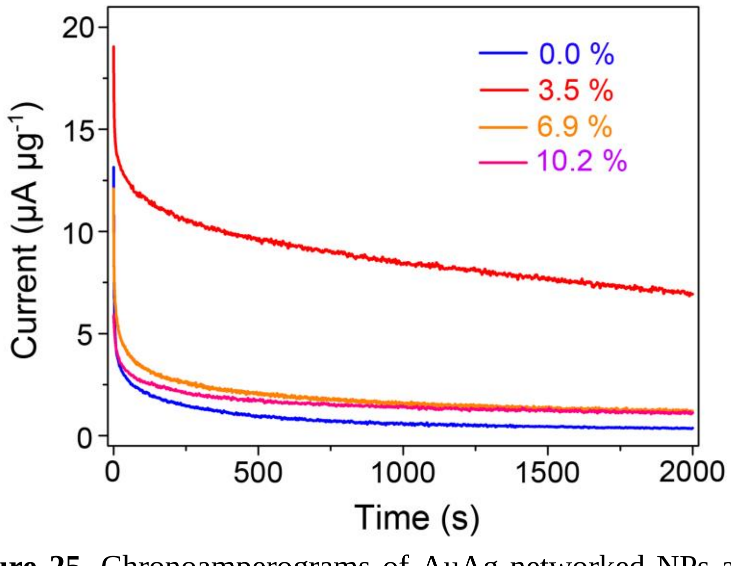

Supplementary Figure 25. Chronoamperograms of AuAg networked NPs at their corresponding peak oxidation potentials, that is, 0.248 V, 0.206 V, 0.198 V, and 0.194 V for Ag composition changing from 0% to 3.5%, 6.9% and 12%, respectively.

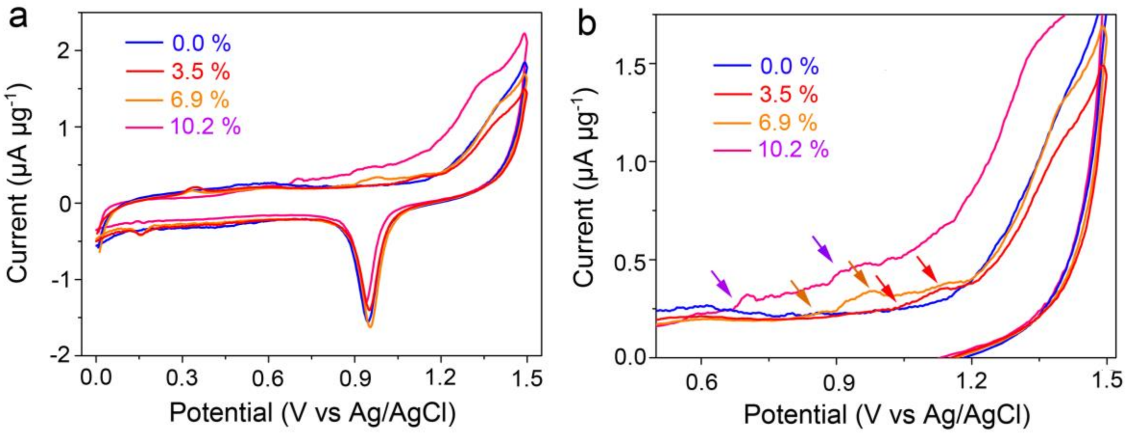

Supplementary Figure 26. (a) Oxide stripping curves of AuAg networked NPs with Ag compositions of 0%, 3.5%, 6.9% and 12%, at a scan rate of 10 mV s^{-1} in 0.5 M H_2SO_4 electrolyte. (b) Magnified image from area of 0.5 V to 1.5 V potential. The arrows note the current of Ag oxidation at onset potential and peak potential.

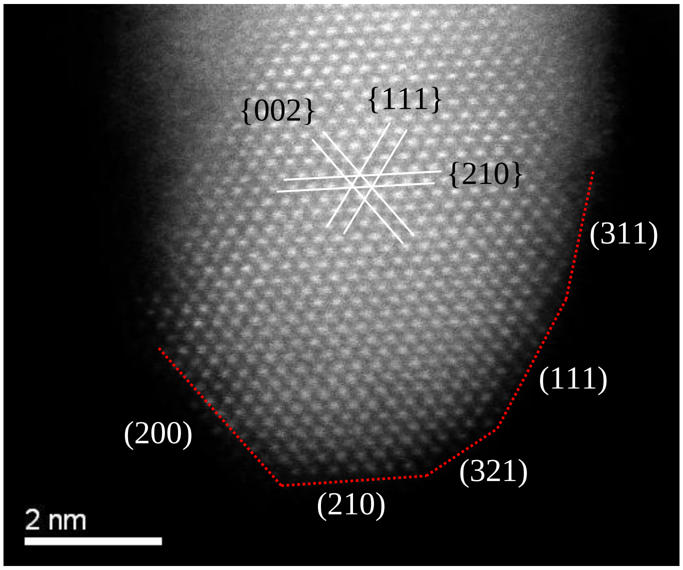

Supplementary Figure 27. Surface atomic structure of AuAg networked NPs. HAADF-STEM images of diverse high-index planes with atomic kinks and steps, showing (311), (321), (210) high-index planes.

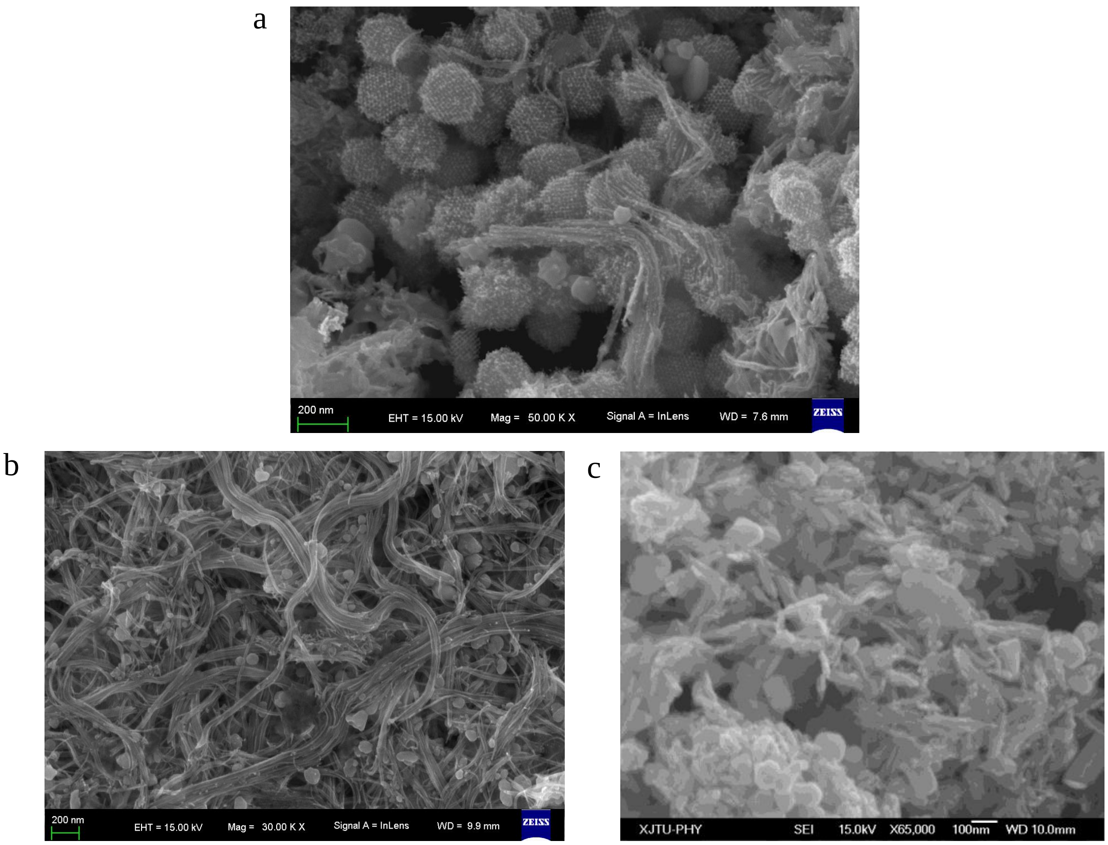

Supplementary Figure 28. The products obtained by a similar reduction process as Figure 6, but without a solvent barrier layer. **a,** the 3D mesoporous Pt networked structure prepared by using a KIT-6 template. **b**, Ag nanowires obtained via an SBA-15 template. **c**, the 3D mesoporous Au nanoparticle superlattice prepared using EP-FDU-12 as the template.

Supplementary Discussion

The durability of catalyst also has been recognized as one of the most important issues to be addressed before the commercialization of polymer electrolyte membrane fuel cells (PEMFCs). Thus, the stabilities of AuAg alloy networks with 6.9% Ag and Au-4 NPs were further evaluated in an accelerated durability test (ADT) by applying linear potential sweeps between -0.15 and 0.55 V (vs Ag/AgCl) at 10 mV s^{-1} in 0.5 M KOH + 2 M CH_3OH aqueous solution. Supplementary Figures 23A and B show the CV curves of AuAg networks and Au-4 NPs catalysts very 500 ADT cycles.

The peak current in the forward CV drops gradually with the increase of CV cycles. The loss of peak current with the increase of CV cycles is plotted and compared in Supplementary Figure 23. After 1000, and 2000 ADT cycles, the current of AuAg network (6.9% Ag) decreases 48.8%, and 61%, respectively. However, the current loss for the Au-4 NPs is 60%, and 84% after 1000, and 2000 ADT cycles, respectively. As reported in previous work, the metal NPs would grow up and aggregate during the durability tests owing to the Ostwald ripening.[2,3] The aggregation and coalescence of metal NPs in the catalysts induced the decrease of specific surface area and catalytic current. The mesoporous AuAg networks are more stable than Au small nanoparticles, and thus show greatly improved durability properties.

By changing the amount of feeding Ag precursor, the composition of Ag in the AuAg networked NPs can be tuned from 3.5% to 6.9% and 12% (determined using inductively coupled plasma atomic emission spectroscopy, ICP-AES). Supplementary Figure 24a shows that the composition of Ag has important effect on the catalytic property. The peak current for the electrocatalytic MOR increases from 13.2 μA μg^{-1} to 19.2 μA μg^{-1} as the composition of Ag change from 0% to 3.5%. Then, with the further increase of Ag amount to 6.9% and 12%, the peak current for the MOR conversely decreases to 12.5 μA μg^{-1} and 6.9 μA μg^{-1}, respectively. Supplementary Figure 24b shows that the AuAg alloy networked NPs possess even lower peak potential and onset potential, compared with pure Au networked NPs. The peak potential and onset potential of AuAg alloy networked NPs decrease a little with Ag amount increase to 6.9% and 12%. From the result, we suggest that the roles of Ag atoms may perform as following. The Ag atoms have no obvious function for the electro-oxidation of methanol, but they can improve the catalytic property of Au atoms by transform electrons to the Au atoms, and thus decrease the activation energy for the MOR. However, although the peak potential and onset potential are decreased by the increase of Ag composition, the total current become lower because more surface area is occupied by Ag atoms, similar with catalytic property of Au-Pt hollow nanourchins.[3]

As shown in Supplementary Figure 25, the long-term electrocatalytic performance and tolerance of AuAg networked NPs are greatly improved, comparing with pure Au NPs. From the chronoamperograms curves of AuAg networked NPs and Au networked NPs at their peak potential for electrocatalytic MOR, we can see that after 2000 s, the currents decrease to 3%, 37%, 10%, and 19% of their peak currents with the composition of Ag being 0%, 3.5%, 6.9%, and 12%, respectively. This result indicates that small amount of Ag element in AuAg alloy nanoparticles can increase the catalytic stability, but more amount of Ag element has no improvement for the stability.

The oxide stripping curves of AuAg networked NPs with Ag compositions of 0%, 3.5%, 6.9% and 12% in Figure S26 show that the electrochemical active surface areas are very close for the AuAg networked NPs with different Ag composition. In the H_2SO_4 solution, the typical redox responses corresponding to the oxidation and reduction of pure Au at the potentials of around 1.3 V and 0.9 V (vs Ag/AgCl). As shown in Supplementary Figure 26, the Au networked NPs (0% Ag) display an oxide stripping curve according well with typical potentials of pure Au oxidation and reduction. Same with the result in references,[4,5] a peak at lower potential correlated with the oxidation of Ag atoms in AuAg alloy is observed for the AuAg networked NPs. With the increase of Ag composition, the onset potential and peak potential for the oxidation of Ag atoms become lower. In the low density of Ag atoms, the Ag atoms mainly are embedded inside of AuAg alloy nanoparticle, thus the oxidation potential may be higher.

Supplementary References

1. Xu, J. B., Zhao. T. S., Yang, W. W. & Shen, S. Y. Effect of surface composition of Pt-Au alloy cathode catalyst on the performance of direct methanol fuel cells. *Inter. J. Hydrogen Energy*, **35,** 8699-8706 (2010).
2. Liang, H.-W.; Cao, X.; Zhou, F.; Cui, C.-H.; Zhang, W.-J.& Yu,S.-H. A free-standing Pt-nanowire membrane as a highly stable electrocatalyst for the oxygen reduction reaction. *Adv. Mater.* **23,** 1467−1471 (2011).
3. You, H. J.; Zhang, F. L.; Liu Z. & Fang, J. X. Free-standing Pt−Au hollow nanourchins with enhanced activityand stability for catalytic methanol oxidation. *ACS Cata.* **4**, 2829-2835 (2014).
4. Pedireddy, S. et al. Nanoporous Gold Bowls: A Kinetic Approach to Control Open Shell Structures and Size-Tunable Lattice Strain for Electrocatalytic Applications. *Small* **12,** 4531-4540 (2016).
5. Tominaga, M., Shimazoe, T., Nagashima, M.,& Taniguchi, I. Composition-activity relationships of carbon electrode-supported bimetallic gold-silver nanoparticles in electrocatalytic oxidation of glucose. *J. Electroanal. Chem.* **615,** 51-61 (2008).
